# Patterns of *in situ* Mineral Colonization by Microorganisms in a ~60°C Deep Continental Subsurface Aquifer

**DOI:** 10.3389/fmicb.2020.536535

**Published:** 2020-11-19

**Authors:** Sean W. Mullin, Greg Wanger, Brittany R. Kruger, Joshua D. Sackett, Scott D. Hamilton-Brehm, Rohit Bhartia, Jan P. Amend, Duane P. Moser, Victoria J. Orphan

**Affiliations:** ^1^Division of Geological and Planetary Sciences, California Institute of Technology, Pasadena, CA, United States; ^2^Jet Propulsion Laboratory, Pasadena, CA, United States; ^3^Division of Hydrologic Sciences, Desert Research Institute, Las Vegas, NV, United States; ^4^Department of Microbiology, Southern Illinois University Carbondale, Carbondale, IL, United States; ^5^Department of Earth Sciences, University of Southern California, Los Angeles, CA, United States

**Keywords:** microbial ecology, deep biosphere, fractured rock, mineral colonization, microbial succession, carbonate, pyrite, methanogenesis

## Abstract

The microbial ecology of the deep biosphere is difficult to characterize, owing in part to sampling challenges and poorly understood response mechanisms to environmental change. Pre-drilled wells, including oil wells or boreholes, offer convenient access, but sampling is frequently limited to the water alone, which may provide only a partial view of the native diversity. Mineral heterogeneity demonstrably affects colonization by deep biosphere microorganisms, but the connections between the mineral-associated and planktonic communities remain unclear. To understand the substrate effects on microbial colonization and the community response to changes in organic carbon, we conducted an 18-month series of *in situ* experiments in a warm (57°C), anoxic, fractured carbonate aquifer at 752 m depth using replicate open, screened cartridges containing different solid substrates, with a proteinaceous organic matter perturbation halfway through this series. Samples from these cartridges were analyzed microscopically and by Illumina (iTag) 16S rRNA gene libraries to characterize changes in mineralogy and the diversity of the colonizing microbial community. The substrate-attached and planktonic communities were significantly different in our data, with some taxa (e.g., Candidate Division KB-1) rare or undetectable in the first fraction and abundant in the other. The substrate-attached community composition also varied significantly with mineralogy, such as with two *Rhodocyclaceae* OTUs, one of which was abundant on carbonate minerals and the other on silicic substrates. Secondary sulfide mineral formation, including iron sulfide framboids, was observed on two sets of incubated carbonates. Notably, microorganisms were attached to the framboids, which were correlated with abundant *Sulfurovum* and *Desulfotomaculum* sp. sequences in our analysis. Upon organic matter perturbation, mineral-associated microbial diversity differences were temporarily masked by the dominance of putative heterotrophic taxa in all samples, including OTUs identified as *Caulobacter*, *Methyloversatilis*, and *Pseudomonas*. Subsequent experimental deployments included a methanogen-dominated stage (*Methanobacteriales* and *Methanomicrobiales*) 6 months after the perturbation and a return to an assemblage similar to the pre-perturbation community after 9 months. Substrate-associated community differences were again significant within these subsequent phases, however, demonstrating the value of *in situ* time course experiments to capture a fraction of the microbial assemblage that is frequently difficult to observe in pre-drilled wells.

## Introduction

The continental deep biosphere represents approximately 5–15% of the biomass on Earth, or about 27–64 Gt of carbon, yet the ecological conditions that govern community structure and activity remain poorly constrained (McMahon and Parnell, 2014; Bar-On et al., 2018; Magnabosco et al., 2018). One factor that complicates our understanding of this vast habitat is that microbial community composition is often predicated on highly heterogeneous local mineralogy and geochemistry (Orcutt et al., 2011; Sylvan et al., 2013; Toner et al., 2013; Jones and Bennett, 2014). Most cells in the deep subsurface are attached to surfaces, not planktonic, with attached cells estimated to outnumber free-living cells by a factor of 10–10^3^ (Wanger et al., 2007; McMahon and Parnell, 2014). Evidence suggests that not only do the mineralogical composition and crystallographic axes affect which microbes attach *via* electrostatic interactions (Yee et al., 2000; Edwards and Rutenberg, 2001), but also that microbes actively weather minerals to access scarce nutrients or by way of their central metabolism (Lovley et al., 1989; Rogers et al., 1998; Caccavo and Das, 2002; Roberts, 2004). These effects can often be challenging to detect *in situ* due to the difficulty of accessing underground fracture networks, and because mineralogy typically varies concurrently with changes in geochemistry, temperature, porosity, and pressure along a depth axis. *In situ* experiments in terrestrial boreholes drilled from the surface are an attractive option because they provide direct access to rock surfaces in the native subsurface biogeochemical context. Subseafloor biosphere studies, such as (Orcutt et al., 2010), which deployed flow-through columns containing mineral colonization substrates of interest, can provide a good model for terrestrial experiments. *In situ* incubation of a variety of mineral substrates enables the study of microorganisms adhering to differing mineral surfaces and further reveals the differences in composition between mineral-adhered and free-living microbial populations. Although drilling in terrestrial environments is comparatively more accessible than in marine systems as described in other studies (Orcutt et al., 2010), such experiments are not without their own challenges (Wilkins et al., 2014). Drilling always carries a risk of contamination, but drilling contamination can be mitigated in pumped or free-flowing boreholes by flushing with local groundwater to remove contaminants and re-establish the indigenous community (Moser et al., 2003; Davidson et al., 2011).

**Figure 1:**
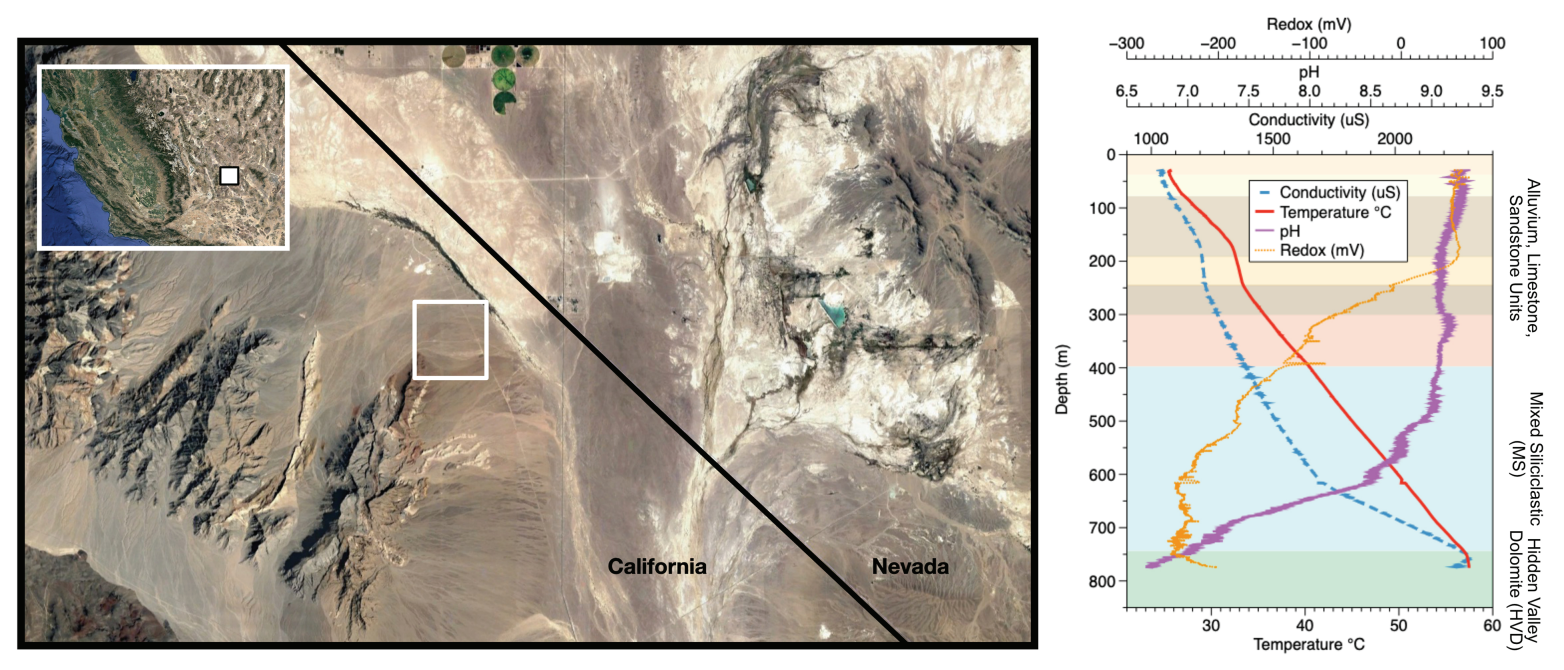
Site location and well profile. (Left) Inyo-BLM 1 is located northeast of Bat Mtn. in the Funeral Mountain Range. Map data: Google, Maxar Technologies. (Right) Geochemical profile of the well overlaid with lithological units as described in Fridrich et al. (2012). The well itself is cased in steel down to 750 m depth, at which point it is open to the Hidden Valley Dolomite (HVD) unit. Geochemical data from the groundwater within the well, which begins at around 28 m depth from surface, are displayed. Although the borehole was originally drilled to 884 m depth, infilling of soft sediments now limits access to approximately 755 m. Map data copyright: attribution of the map data follows Google Earth guidelines.

Describing the ecology of the subsurface can also prove difficult because of the extremely slow growth and metabolism of many resident microorganisms. *In situ* doubling times for virtually all deep biosphere organisms are unknown and most studies rely on biomass turnover rates instead, calculated from cell-specific activity measurements and amino acid racemization studies (Kieft and Phelps, 1997; Morono et al., 2011; Onstott et al., 2014; Braun et al., 2017; Trembath-Reichert et al., 2017). These studies suggest biomass turnover rates in the deep biosphere range from several months to thousands of years. In fact, in the most oligotrophic environments such as the ocean gyre subseafloor, reactive transport models indicate that resident cells, if they are growing, either have energy requirements below the reported power usage minimum required for cellular maintenance (LaRowe and Amend, 2015) or are not growing at all, but slowly dying (Bradley et al., 2018). In less austere environments, such as the coastal marine subsurface or the terrestrial deep biosphere, microbial community analyses do not indicate that these environments only host poorly-adapted surface organisms. Instead, these environments are home to their own endemic, uncultivated, and previously unknown organisms such as the aptly named Deep Sea Archaeal Group, novel branches of Chloroflexi, or Candidate Phyla (Takai et al., 2001; Inagaki et al., 2006; Jørgensen and Boetius, 2007; Osburn et al., 2014). The terrestrial deep biosphere, accessed *via* boreholes or mines, has proven especially fruitful for the discovery of novel microbial diversity, including from the site in this study (Figure 1; Chivian et al., 2008; Sahl et al., 2008; Teske and Sørensen, 2008; Breuker et al., 2011; Osburn et al., 2014; Momper et al., 2017, 2018; Sackett, 2018; Sackett et al., 2019). Little is known, however, about the changes in deep subsurface systems of yearly or longer timescales. It may be possible that deep biosphere organisms persist with little to no growth until a rare nutrient or energy delivery, perhaps mediated by tectonic disturbance (Riedinger et al., 2015). Controlled substrate addition experiments performed *in situ* offer a novel experimental approach to understand the influence of transient energy influx on deep biosphere community structure and help constrain the energy requirements and maximum metabolic rates of these organisms.

Here, we describe an 18-month series of five *in situ* microbial colonization experiments to characterize and constrain the microbial community and activity of a warm, continental biosphere from a ~775 m-deep borehole (Inyo-BLM 1) hosted in a fractured, Paleozoic carbonate beneath the Mojave Desert, CA, United States (Figure 1). We demonstrate that not only is the mineral-hosted microbial community significantly different from the planktonic community, but that mineral-hosted communities vary based on the mineralogy. We also demonstrate that a controlled organic substrate addition strongly affected the *in situ* microbial community composition and revealed subsurface taxa that were poised to take advantage of introduced complex organic matter.

## Materials and Methods

### Field Site

The Inyo-BLM 1 well was completed in 2007 to a total depth of 884 m below land surface (mbls) by The Hydrodynamics Group, LLC (Edmonds, WA) on behalf of Inyo County, California (Belcher et al., 2009; Bredehoeft and King, 2010). The well pad is on the eastern flank of the Funeral Mountains in the Amargosa Valley, proximal to the California-Nevada border near Death Valley National Park (36°24'04.19N/116°28'06.58W). Inyo-BLM 1 was drilled to test hypotheses concerning deep groundwater flow across the Funeral Mountains and intersects a variety of lithologies (e.g., sandstone and tuff), ultimately encountering Hidden Valley Dolomite (HVD; Silurian-Devonian, 423-393 Ma, in age) at 744 mbls (Fridrich et al., 2012), which is associated with the Lower Carbonate Aquifer (LCA), a warm, fractured rock aquifer in the discharge zone of the Death Valley Regional Flow System (Belcher et al., 2001; Bredehoeft et al., 2008; Figure 1). Inyo-BLM 1 was pumped for development and hydrologic testing in both 2007 and 2011 by pumping out approximately 1.6 million liters of water (~100 times total volume of groundwater in Inyo-BLM 1) and then allowed to re-equilibrate with the surrounding anoxic aquifer. Other than these two pumping events in 2007 and 2011, the hole sat undisturbed apart from continuous monitoring of the static water level by the National Park Service to measure water table elevation until our sampling began in 2014. Although originally drilled to a total depth of 884 m, at the time of sampling, Inyo-BLM 1 was only accessible to 755 mbls, presumably due to collapse of unstable rock below the casing. Inyo-BLM 1 is continuously cased in unscreened low-carbon steel and cemented in a step-down manner (Bredehoeft et al., 2008); caliper measurements were completed in 2015 showing that the inner diameter decreases from 14.5–15 cm diameter (0–610 mbls) to 8.4–8.6 cm diameter (610–750 mbls), with the final 5 m open to the host rock and the warm (57°C) reducing fluids of the LCA. The borehole passes through several lithologies, including sandstone, siltstone, and mixed siliciclastic and tuff lake sediments; however, the open hole section is entirely hosted within the HVD within the Lost Burro Formation, roughly Silurian-Devonian (423–393 Ma) in age (Fridrich et al., 2012). Although the Inyo-BLM 1 drill pad is located hundreds of meters above the valley floor (694 m above sea level), hydrostatic pressure maintains the groundwater at approximately 28 m below the surface (USGS, 2018). A study by Thomas et al. (2013) indicated that although this hole likely shares the same hydrological recharge area as other wells in the area, including Ash Meadows springs, it is likely primarily sourced from a deeper and older portion of aquifer. A thermal flowmeter was deployed on August 21, 2015 to test for flow within the well, but none was detected (i.e., <0.08 L per min; not shown).

### *In situ* Experimental Incubations

To characterize the rock-associated microbial community, several sterilized solid substrates of defined or native materials were incubated *in situ* within polytetrafluoroethylene (PTFE) open-ended cartridges (2 cm inner diameter, approximately 18 cm long) screened with 1 mm nylon mesh. Cartridges were filled with pre-characterized substrates, which consisted of either mineral standards or cuttings of the appropriate lithologic units obtained during the drilling process and archived at the USGS Core Library and Data Center (Mercury, NV). Incubated standard substrates included glass wool (Sigma Aldrich #18421), dolomite (Ward’s Science #470025-558), pyrite (Ward’s Science #470025-736), optically clear calcite (Ward’s Science 470025-522), pyrrhotite (Ward’s 470025-750), and 3 mm diameter low-carbon steel ball bearings (McMaster-Carr 96455K49) as a proxy for the well casing. Natural lithologies tested included HVD, a fine-grained dark-gray dolomite with nodular chert, and mixed siliciclastic lake sediments and tuff (MS). Crushed minerals ranged in size from 2 to 5 mm. *In situ* incubations containing dolomite, HVD, calcite, or MS were always conducted in duplicate (*n* = 2 cartridges) for each time point. Cartridges were assembled into bundles of three, secured with 200-test Dyneema™ high-strength line (Supplementary Figure S2). Duplicate cartridges were always assembled into separate bundles. Three 5 mm PTFE rods acted as a cage for the bundles. The entire assembly was autoclaved and maintained in a sterile bag until deployment. During the deployment of the cartridges, an A-frame was constructed over the well cap, and the cartridge string was lowered into the well affixed to both 200-test and 500-test Dyneema™ fishing line as a failsafe. At regular intervals (~75–100 m) a series of polyurethane (PUR) foam blocks (Identi-plug™, Fisher Scientific Inc.) were attached to the fishing line with nylon cable ties (McMaster-Carr 70215K96) following Imachi et al. (2011). The incubation package was suspended in the open hole portion of the borehole at ~752 m depth, within the HVD unit, for periods of 3–6 months before retrieval with five total incubations over 18 months (Table 1). Upon retrieval, cartridges were stored at ambient temperature in oxygen impermeable mylar bags with sterile oxygen scrubbing packets (Oxy-Sorb; Harrisburg, NC) until they could be disassembled and subsampled under sterile conditions in a laboratory anaerobic chamber the following day. Subsamples were frozen at −80°C for DNA analysis or fixed individually in electron microscopy-grade 2% paraformaldehyde or 2.5% glutaraldehyde (Electron Microscopy Sciences; Hatfield, PA) for microscopy. The dates and mineral substrates included for each deployment are provided in Table 1.

**Table 1:** Contents and timetable of *in situ* incubations.

Incubation number	Date deployed	Date recovered	Total days *in situ*	Mineral cartridges	Notes
1	11/3/14	2/5/15	94	Glass wool, Ward’s calcite, Ward’s dolomite, Hidden Valley Dolomite (HVD), Tuff and mixed siliciclastic lake sediments (MS), Low-carbon steel	
2	2/5/15	8/21/15	197	Glass wool, Ward’s calcite, Ward’s dolomite, HVD, MS, Low-carbon steel	Well-logging and geochemical analysis performed after recovery.
3	8/23/15	11/5/15	74	Glass wool, Ward’s dolomite, HVD, MS, Low-carbon steel, Ward’s pyrite, Ward’s pyrrhotite	Five natural sea sponges attached to line above minerals.
4	11/5/15	2/4/16	91	Glass wool, Ward’s calcite, Ward’s dolomite, HVD, MS, Low-carbon steel	Only one natural sponge from Inc. 3 recovered. Others were degraded and fell off the line.
5	2/4/16	5/11/16	97	Glass wool, Ward’s calcite, Ward’s dolomite, HVD, MS, Low-carbon steel	

### Groundwater Geochemistry and Microbiology

In the period following retrieval of Incubation 2 and prior to deployment of Incubation 3, the borehole was chemically and physically characterized using calipers to measure the interior diameter, a thermal flow meter, and a QL40-Ocean Idronaut wellbore logging multiprobe (Idronaut, S.R.L.; Brugherio, Italy) that concurrently measures temperature, pH, relative oxygen reduction potential (ORP), and conductivity over the entire depth profile. Several liters of water from 579 to 752 m were also collected at this time by successive collection at ambient pressure with gas-tight discrete samplers (“bailers,” Comprobe, Inc.; Fort Worth, TX) for extensive aqueous geochemical and dissolved gas characterization, with the shallower depth sampled first to prevent mixing the water column. Dissolved metals and ions were quantified by ACZ Laboratories (Steamboat, CO), and dissolved organic carbon (DOC), particulate organic carbon (POC), and total organic carbon (TOC) were quantified by Anatek Labs (Spokane, WA), both of which are accredited environmental testing facilities. The methodologies and standard error values for these analyses are included in Supplementary Table S2, with the error values from ACZ Laboratories generated from duplicate analyses of a standard and the error values from Anatek Labs from duplicate sample analyses. Samples from two depths were analyzed (579 and 752 mbls). Sulfide was measured using the methylene blue method with a field detection kit (detection limit ~0.02 mM; ChemMet, LLC.; Buffalo, New York), following the manufacturer’s protocol. We also conducted a qualitative *in situ* test for dissolved sulfide using silver nitrate-coated film, following (Fike et al., 2017). The environmental detection limit of this method has not been well-quantified, but data suggests that 20 min of exposure to a 0.9 mM sulfide solution is more than sufficient for visualization. The film was attached to the discrete water sampler, lowered to 752 m depth in the uncased portion of the borehole, left stationary for 2 min, and then recovered to the surface, for a total time of ~45 min.

To measure dissolved gases in the well waters (Supplementary Table S2), sterilized 160 ml serum bottles were prepared with 50 μl saturated (0.07 g/ml) HgCl_2_ solution and capped with butyl rubber stoppers. The HgCl_2_ solution was then evaporated away to dryness under constant vacuum, leaving only the 3.5 mg of HgCl_2_ behind. Water samples were collected by draining the first ~50 ml fluid fractions from three separate 750 m depth water samples through platinum-cured silicone tubing (Masterflex LS-24) and a 25 G needle into the pre-evacuated serum bottles. The filled vials were stored upside down until their analysis within 8 weeks. Headspace concentrations of H_2_, O_2_, N_2_, CO_2_, CO, ethane, and propane were measured using a Shimadzu GC-2014ATF headspace gas chromatograph (GC) equipped with a Haysep 80/100 (5 m) and MS-5A 60/80 (2.5 m) molecular sieve columns and TCD and FID detectors. Samples were measured twice (relative percent differences listed in Supplementary Table S2) and were compared against a 1% standard mixture (Restek; Bellefonte, PA). See (Rowe et al., 2017) for details.

Microbial cell counts of the groundwater were performed in two ways (live and fixed) on fluids from 579 to 752 mbls collected August 20–21, 2015. Samples collected from three separate casts for each depth were counted in each way. Live samples were stored at 4°C until they were processed (August 21). Samples were also fixed in the field with 1% paraformaldehyde and stored at 4°C until they were processed on August 23, 2015. Live samples were counted with a Petroff-Hausser counting chamber, and fixed samples were stained with 4',6-diamidino-2-phenylindole (DAPI) and counted on fluorescence microscope (Olympus BX51, Olympus Scientific Solutions; Waltham, MA) at 1,000× magnification (Supplementary Table S3).

### Amendment of Complex Organic Matter *in situ*

As part of a separate experiment, five natural sea sponges (Constantia; Hawthorne, CA) were attached to the line directly above the mineral cartridges (~752 mbls) during Incubation 3 as well as higher up on the suspension line at 579 mbls. All sponges from the 752 mbls set, closest to the experimental cartridges, were consumed by microbial activity by the time of recovery; one sponge at 579 mbls remained at the end of the experiment, however, this sponge was noticeably degraded with a gelatinous consistency. This degraded material was salvaged from the line and frozen for microbial community analysis.

To approximate the amount of organic carbon and nitrogen introduced into the deep aquifer and potentially consumed by *in situ* microorganisms, the composition of the sponges was estimated. The purchased sea sponges deployed in Incubation 3 belong to the family Dictyoceratida, which have minimal or no calcareous spicules in their skeleton and are primarily composed of spongin, a collagenous halogenated glycoprotein with high thermal stability up to ~360°C (Ehrlich et al., 2003, 2010; Green et al., 2003; Norman et al., 2016). Prior to deployment, the sponges were washed, bleached with hydrogen peroxide and rinsed, and sterilized by autoclaving for 30 min. Spongin’s chemical formula is unknown, but the carbon and nitrogen content has been quantified (carbon = 47.44% and nitrogen = 16.15%; Gamgee, 1880). Each sponge was on average 0.37 g, and based on the observation that all sponges from the region proximal to the Incubations appeared to be entirely degraded by the time Incubation 3 was recovered, we estimate the total carbon and nitrogen added to the system in the proximity of the mineral cartridges was ~0.88 and ~0.30 g, respectively.

We developed a rough diffusion-based model to approximate the time for degradation and dispersal of the sponge-derived organic matter. As no vertical flow was detected within the well, we assumed dispersal of the solubilized, degraded sponge substrate occurred through molecular diffusion. This model was simplified to a one-dimensional diffusion in two directions, described by the thin-film (Gaussian) solution to Fick’s Second Law:

$$C\left(x,t\right)=\frac{N}{2\sqrt{\pi Dt}}*e^{\frac{-x^{2}}{4Dt}}$$

where *C*(*x,t*) is the concentration of the molecule of interest at position *x* and time *t*, *N* is the moles of the molecule per unit area at *x_0_* and *t_0_* (12.58 mol C/m^2^, given well diameter of ~8.6 cm), and *D* is the diffusion coefficient (Mehrer, 2007). We bounded the time range for the diffusion of the sponge nutrients by setting *t_0_* as either the moment the sponges are in place or halfway through Incubation 3, which assumes that the sponge organics do not diffuse for the first 37 days and then become completely diffusible after that time (37 ≤ *t_0_* ≤ 74). Diffusion coefficients, *D*, are normally only determined empirically, which was not possible here. As an approximation, we used the *D* for lactose at 55°*C*: 1.06 × 10^−9^ m^2^/s (Ribeiro et al., 2006). Based on the cartridge bundle dimensions, which extended from approximately 0.25–2.0 m from the sponges, and accounting for the well casing with an average diameter of 0.086 m, we estimate 10.6 L of water surrounded the cartridge bundle. Using all of these parameters, we determined a rough estimate of the total moles of organic carbon released into the water surrounding the cartridges by integrating *C(x)* over the distance at either *t* = 37 days or *t* = 74 days (depending on which *t_0_* is used) and multiplying by the volume, which translates to values in the range of 160 micro µ mol carbon to 3.01 mmol carbon. We thus assume that retrieval of the experiment would mix any remaining dissolved carbon species upward into the remaining 733 m of the water column; diffusion downward from these distances over the remaining time scale of our experiments is trivial.

### Abiotic H_2_ Production From Steel

Hydrogen production from the steel alloy used in the well casing was verified (see Supplementary Figure S5) by adding one of several Fe-bearing substrates to a defined medium (Supplementary Table S1) designed to approximate well fluids at 579 mbls as determined by the geochemistry suite from 8/21/15 described above (Supplementary Table S2). The substrates tested for hydrogen production were low-carbon steel analogous to the well casing material (McMaster-Carr 96455K49), 304 stainless steel (McMaster-Carr 9291K43), 316 stainless steel (McMaster-Carr 96415K71), and hematite (Ward’s Science 470025). Three replicate vials were measured for each substrate. For steel incubations, three steel ball bearings were added to the sterilized defined medium, and for hematite, 2–5 mm crushed rock was weighed to be volumetrically equivalent to the steel. The headspace of each tube was measured with the same headspace gas chromatograph program as was used to measure the natural samples, as described above.

### DNA Sequencing

DNA was extracted from environmental samples with the Qiagen PowerSoil DNA Extraction Kit (Qiagen; Hilden, Germany; 12888) according to the kit protocol except for the following changes during the lysis step, performed with the intention of extracting DNA from as much of the community as possible. Following the addition of 0.25–0.5 g of sample to the lysis tubes, the tubes were flash frozen in liquid nitrogen. The samples were thawed at room temperature and then vortexed for 1 min. The lysis buffer (30 μl C1 buffer) was then added and the samples were incubated in a 65°C water bath for 30 min, with each sample vortexed for 1 min every 10 min. The samples were then placed in a bead beater (FastPrep FP120, Thermo Fisher Scientific; Waltham, MA) at 5.5 m/s speed for 45 s. The samples were then centrifuged at 10,000 × *g* for 30 s and processed according to the rest of the manufacturer’s instructions. To keep extraction conditions consistent, filtered and unfiltered water samples were extracted with the same kit. Filtered water samples consisted of 250 ml of raw water passed through a 0.22 μm PES filter (UX-06730-43, Thermo Scientific; Waltham, MA). Approximately 0.25 g of this filter was then processed as above. To prevent detection bias against cells smaller than 0.22 μm, unfiltered water was also extracted by adding 250 μl of Inyo-BLM 1 water to the PowerSoil lysis tubes and then processed with the same alterations to the extraction protocol.

Microbial diversity was assessed by Illumina iTag sequencing of the V4 region of the 16S rRNA gene, using a protocol recommended by the Earth Microbiome Project (Caporaso et al., 2012) and sequenced with Illumina Tag sequencing by Laragen, Inc. (Los Angeles, CA) as previously described (Caporaso et al., 2012; Case et al., 2015). The V4 region of the 16S rRNA gene was amplified using degenerate 515F and 806R primer pairs (5'-GTGCCAGCMGCCGCGGTAA-3' and 5'-GGACTACHVGGGTWTCTAAT-3'). Each sample was amplified in duplicate and the resulting amplicons were pooled. Each reaction was 15 μl total: 7.5 μl Q5 Hot Start High-Fidelity 2x Master Mix (New England BioLabs, Inc.), 5 μl DNAse-free water, 0.75 μl of each primer at 10 μM, and 1 μl of gDNA extract (~0.5 ng/μl) from the sample. Reactions were held at 98°C for 2 min to denature the DNA, with amplification proceeding for 30 cycles at 98°C for 10 s, 54°C for 20 s, 72°C for 20 s; a final extension of 72°C for 2 min was added for complete amplification. These PCR products are checked for purity *via* gel electrophoresis and the DNA concentration quantified by Qubit HS (Bio-Rad, Inc.) before they are barcoded for sequencing. Barcoding primers include the original primer sequence as well as linkers, adapters, and on the reverse primer, a unique barcode as described previously (Mason et al., 2015). The reaction used 5 μl of the 16S rRNA DNA product, 12.5 μl of Q5 Host Start Master Mix, 1.25 μl of 10 μM forward primer, 5 μl of 2.5 μM reverse primer, and 1.25 μl DNAse-free water. Reaction temperatures were the same as above.

Extraction controls and no-template controls were included in every sequencing run; controls were amplified with 45 reaction cycles in the initial PCR reaction to produce a quantifiable PCR product. Autoclaved control minerals that were not incubated *in situ* were also extracted and sequenced after 45 cycles of amplification (control minerals amplified 30 times did not produce a detectable gel electrophoresis band) to control for DNA that may have survived sterilization. Supplementary Figure S1 is a comparison of mineral controls vs. incubated minerals. Sequencing sample metadata is listed in Supplementary Table S5, and the resulting OTU table with the associated sequence and taxonomic identification is listed in Supplementary Table S6. Raw sequenced data is available at the NCBI SRA database with accession number PRJNA605006.

### Illumina Amplicon Sequence Analysis

In-house amplicon processing was completed with QIIME 1.8.0 and included joining paired-ends, quality trimming, 99% OTU clustering, singleton removal, and 0.01% relative abundance threshold removal (Caporaso et al., 2010). Remaining OTUs were then manually checked against sequenced extraction and no-template controls and non-incubated mineral controls to remove contaminating sequences. OTUs with relative abundance ≥0.05% in the controls were removed from the dataset. A total of 118 OTUs were removed during this step, leaving 520 OTUs remaining after all filtering steps. The average number of reads per sample remaining after quality-score filtering and removal of singletons, threshold filtering, and manual contaminant filtering was 23,742, with a range of 5,287–47,225. The Shannon diversity index and Chao1 richness estimate were computed for each sample and are listed in Supplementary Table S5. Although some studies examining high-throughput sequencing data rarefy each sample to the lowest sequence sample, we chose not to rarefy due to several recent studies that indicate that this transformation is not appropriate and can impede detection of differential abundance (McMurdie and Holmes, 2014; Weiss et al., 2017). These studies indicate that transforming sequence counts into relative abundance is more effective at detecting true differential abundance and does not require discarding valid data. Taxonomic assignments for each OTU were generated by comparing against the Silva 138 database (90% cut-off). Further statistical analyses, including non-metric multidimensional scaling (NMDS), analysis of similarity (ANOSIM), similarity percentage (SIMPER), and one-way ANOVA were conducted in R (R Core Team, 2014) with the *vegan* ecological statistics package. NMDS analyses were conducted after performing a fourth root transformation to the relative abundance data, following studies indicating that this is a simple but effective way to evaluate differential abundance in microbial communities (McMurdie and Holmes, 2014; Weiss et al., 2017). These analyses construct *de novo* ordinations based on ranked dissimilarities, and the fit of the ordination is measured by the “stress.” In general, stress values below 0.2 are acceptable, and all NMDS ordinations presented here have stress <0.2. In our ANOSIM tests, *p* < 0.05 were considered significant. ANOSIM R values indicate the strength of dissimilarity between groups from 0 to a maximum of 1. SIMPER tests were applied in R to identify specific OTUs with different relative abundances between sample groups, such as between mineral types or between individual incubations. OTUs identified by SIMPER that contributed most to sample differentiation were included for presentation, with the significance of the differential abundance of individual OTUs assessed by ANOVA. Venn diagrams of community overlap (Supplementary Figure S6) were made with Venny (Oliveros, 2007–2015).

### Imaging of *in situ* Incubated Minerals

All mineral samples were imaged both pre- and post-incubation to record any incubation-associated mineralogical changes. Glutaraldehyde-fixed mineral samples for scanning electron microscopy (SEM) were dehydrated with an ethanol dehydration series (25, 50, 75, 90, and 100%; each step 30 min), then critical-point dried with liquid CO_2_ to preserve cell morphology. Briefly, this involves washing out the ethanol with liquid CO_2_ in a pressure chamber kept cold with an ice water bath, then raising the temperature to 31°C and adjusting the internal pressure to 74 bar (the critical point of CO_2_), and then slowly bleeding away the remaining CO_2_. The samples were mounted on aluminum stubs with carbon tape and sputter-coated with 10 nm of 80:20 Platinum:Palladium to minimize charging effects. SEM was carried out on a Hitachi SU3500 microscope (Hitachi High-Technologies; Tokyo, Japan) equipped with an Oxford X-Max 150 energy dispersive x-ray spectroscopy (EDX) unit (Oxford Instruments; Abingdon, United Kingdom).

### Mineral Composition

The calcite and dolomite standards, along with the HVD and MS natural samples, were first powdered manually with an aluminum oxide mortar and pestle. The resulting powder was then analyzed by X-Ray Fluorescence (XRF) with an INAM Expert 3L tabletop XRF under helium purge with a Ti target using INAM’s built-in fundamental parameters software. Data were acquired over 60 s with 15 kV excitation energy for light elements, followed by an additional 60 s with 45 kV excitation voltage for heavier elements. The resulting comparative elemental analyses (as the corresponding elemental oxides) are listed in Supplementary Table S4.

## Results

### Well Logging and Geochemistry

Well logging and geochemical analyses of Inyo-BLM 1 in August 2015 were consistent with well logging data collected in 2011. Over the 18-month period of our experiments, the water level in the well was 28.7 m ± 0.03 mbls (USGS, 2018). The temperature increased by 4.8°C per 100 m from 25.7°C at the surface to 57°C at the bottom of the well (Figure 1). The pH of the fluids down to 500 mbls was slightly alkaline (9.0–9.2), transitioning to circumneutral conditions (6.9) in the uncased portion of the well (depth > 750 mbls). Water samples were collected at 752 mbls (in the uncased portion) and at 579 mbls (Supplementary Table S2). Both samples showed brackish conditions [9.7 and 9.4 mM Na^+^ and 1.5 and 1.4 mM Cl^−^, respectively (222, 215, 52.4, 49.2 ppm); Supplementary Table S2]. Ferrous iron concentrations increased by 60% (0.90–1.4 μM; 0.05–0.08 ppm) and sulfate levels were three times higher (reaching 1.6 μM) in the uncased portion of the hole at 752 mbls relative to 579 mbls (Supplementary Table S2). Calcium, magnesium, and silica concentrations also increased below the casing. Sulfide was not detected by an onsite Cline assay, and a qualitative *in situ* test for dissolved sulfide using the silver nitrate-coated film method also indicated that sulfide concentrations were below detection (Fike et al., 2017). Continuous ORP data obtained with the Idronaut well-logging tool showed a dramatic drop in potential at about 200 mbls (or 172 m below water level), indicative of anoxia (Figure 1). Within the open hole portion at the bottom, the ORP drifts toward positive values, but these types of probes are sensitive to flocculent material in the water. We cannot determine whether the drift is due to an actual increase in the ORP or the presence of suspended particles at depth. Upon recovery of Incubation 2 on 8/21/15, average planktonic cell counts at 752 mbls were 4.1 × 10^6^ cells per ml ± 2.8 × 10^6^, the large standard deviation owing to the tendency of cells to form small clumps (Supplementary Table S3).

### H_2_ Production by Anaerobic Corrosion of Steel

Analyses of dissolved gases in water samples obtained from 579 to 752 mbls in Inyo-BLM 1 immediately preceding the carbon amendment (start of Incubation 3) revealed up to 30-fold more dissolved hydrogen in the cased section of the well relative to the open section (200 vs. 7 μM). The increase in hydrogen co-occurred with a 10-fold increase in dissolved methane (100 vs. 10 μM; Supplementary Table S2). By contrast, the fluids at 752 mbls contained elevated dissolved CO_2_, consistent with a carbonate-hosted aquifer.

To test whether the steel casing could have produced hydrogen (*via* the Schikorr reaction), we incubated three alloys of steel as well as hematite (Fe_2_O_3_) in sterile media mimicking the water chemistry in the borehole (Schikorr, 1929; Linnenbom, 1958; Ma et al., 2013). These experiments verified that low-carbon steel similar to that used in the well casing (and used in our experimental cartridges) produced significantly more H_2_ than stainless steel alloys or hematite (Supplementary Figure S4). Within 13 days, the low-carbon steel produced 14.3 μmol H_2,_ approximately 70-fold more hydrogen than the 304 stainless steel, and 700-fold more hydrogen than the 316 stainless steel alloy or hematite, which produced barely detectable hydrogen peaks during chromatography analysis.

### Mineralogical Analysis and *in situ* Secondary Iron Sulfide Formation

Unincubated calcite, dolomite, HVD, and MS substrates were analyzed by XRF spectroscopy to compare their compositions (Supplementary Table S4). The elemental composition of the substrates was consistent with their mineralogical identification and in the case of the natural HVD and MS samples, with previous geological descriptions (Fridrich et al., 2012). The HVD and dolomite standards differed in their trace element composition, with the dolomite standard higher in iron and manganese and the HVD higher in silicon. The MS sample was consistent with a mixture of potassium and calcium aluminosilicates with a wide array of trace elements detected, including significant amounts of iron, manganese, and titanium.

**Figure 2:**
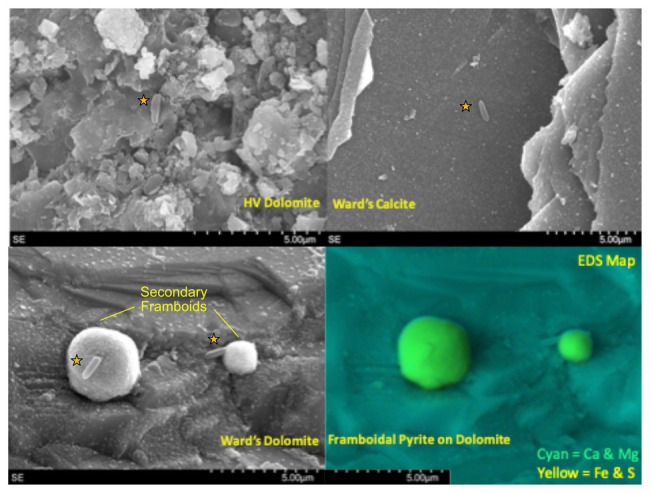
Scanning electron micrograph (SEM) and energy dispersive x-ray spectroscopy (EDX) overlays of carbonates from Inc. 1, with microorganisms marked with orange stars. (Top Left) Natural dolomite sample (HVD) with putative cell in center. The texture of the rock presented difficulties in distinguishing cells from surroundings. (Top Right) Although calcite samples were the easiest to image, few cells and no iron sulfide framboids were visible. (Bottom Left) Cells attached to secondary framboidal structures on the dolomite standard. (Bottom right) EDX map of bottom left panel which confirms that secondary framboids are iron sulfide.

All mineral substrates were imaged by SEM pre- and post-incubation in Inyo-BLM 1. Apart from microbial attachment, the majority of incubated minerals displayed no obvious mineralogical alteration over the course of their incubation. Notable exceptions included the two dolomitic rocks (dolomite standard and native HVD) recovered from the 3-month long Incubation 1 (2/5/15), which showed widespread precipitation of 1–5 μm diameter secondary minerals with framboidal structure (~10 per 100 μm square viewing area); EDX characterized these secondary minerals as iron sulfides (Figure 2). While large anhedral pyrite crystals were observed in both the standard dolomite and HVD samples prior to deployment, nothing resembling the framboidal precipitates were observed on any samples prior to deployment, indicating that they formed *in situ*. These secondary minerals were typically associated with 1–3 microbial cells. Sulfide framboid generation was only detected in the first set of incubations, prior to the organic matter amendment experiment. Sulfide mineral formation was also observed on minerals from the 6-month long Incubation 2 (recovered 8/21/15), in this case producing scattered zinc sulfide spheroids, rather than iron sulfide framboids, but spheroid-attached cells were not observed. In both cases, secondary mineral formation was only detected on the dolomitic minerals (HVD and Ward’s dolomite, *n* = 8) across two separate incubations and not on any glass wool, MS, low-carbon steel, or calcite minerals incubated in parallel (*n* = 14). Incubations 1 and 2 occurred prior to the organic matter addition in Incubation 3 on 8/23/15, and no further secondary mineral formation was observed *via* SEM following the organic matter addition.

### Microbial Community Analyses

After removing potential contaminants found in the sequencing controls, analysis of the Illumina 16S rRNA gene amplicons (iTag) of all samples taken from the site, including incubated mineral substrates from cartridges suspended at 752 mbls, PUR foam blocks from the suspension line, and water from 579 to 752 mbls, revealed a system dominated by Firmicutes, Nitrospira, Alphaproteobacteria and Betaproteobacteria, and Candidate Division KB-1 (also known as Candidate Division Acetothermia), with a moderate relative abundance of Chloroflexi, Epsilonproteobacteria, and Euryarchaeota, including two putative methanogens of the Methanobacteriales and an OTU related to the South African Gold Mine Group of Thermoplasmata (see Supplementary Table S6). The samples demonstrated low diversity overall; the maximum observed Shannon Index was 4.03, and the maximum Chao1 richness was 360. Reflecting the low diversity, the dominant OTU in 60 of the 71 samples represented >15% of the total community for that sample. Across all samples and incubations, two broad groups appeared when visualizing the microbial community by NMDS: one group consisting of water (including unfiltered and filtered water) and the other consisting of solid substrates, including minerals and glass (Supplementary Figure S4). PUR samples occupied ordination space between water samples and mineral samples but contained no unique OTUs. Instead, the PUR-colonized community represented a mixture of planktonic and surface-attached microorganisms (Supplementary Figure S5).

Analysis of similarity (ANOSIM) analyses of the samples were conducted to assess which parameters correlated most strongly with community variance; ANOSIM R values range from 0 to 1 with increasing correlation. When examined across the entire dataset, the most important parameter in the variation between samples was the Incubation number (*R* = 0.4535, *p* = 0.001), with type of substrate showing secondary importance for community differences (*R* = 0.2667, *p* = 0.001). Interestingly, carbonate samples, which included calcite, dolomite, and HVD, grouped together in all but one incubation (Incubation 1). Across all incubations, the communities colonizing carbonate minerals were not significantly different from each other (*p* = 0.558). We also assessed the difference between filtered and unfiltered water samples and found no significant difference (*p* = 0.2). Although we had originally hypothesized that the community colonizing the PUR samples would group with the water samples, these samples were significantly different from the water samples (*p* = 0.001). Based on the ANOSIM analyses and the similarity between the sample types, we therefore grouped the calcite, HVD, and dolomite samples into one “carbonate” bin, and the water samples (filtered and unfiltered) into one “background” bin, which increased the contribution of substrate to the variance (*R* = 0.3235, *p* = 0.001). The apparent dominance of the incubation reference number in describing the community structure was largely due to the organic matter amendment during Incubation 3. On an individual Incubation basis, colonizing substrate (e.g., carbonate, MS, and silica glass wool) was generally a strong determinant of variability (Figure 4). The ANOSIM R statistic and significance values for mineralogical variation when comparing within individual Incubations were as follows: Incubation 1 (*R* = 0.9733, *p* = 0.005), Incubation 2 (*R* = 0.5774, *p* = 0.006), Incubation 3 (not significant, *p* = 0.053), Incubation 4 (*R* = 0.6406, *p* = 0.026), and Incubation 5 (*R* = 0.3900, *p* = 0.026). ANOSIM analysis comparing Incubations 1 and 2 (which occurred prior to organic amendment) revealed the strong effect of substrate type (using the carbonate bin) over Incubation (*R* = 0.7669, *p* = 0.001 vs. *R* = 0.2167, *p* = 0.006). When carbonate minerals in these two incubations were treated as independent, the effect was still significant but less impactful (*R* = 0.6764, *p* = 0.001).

**Figure 3:**
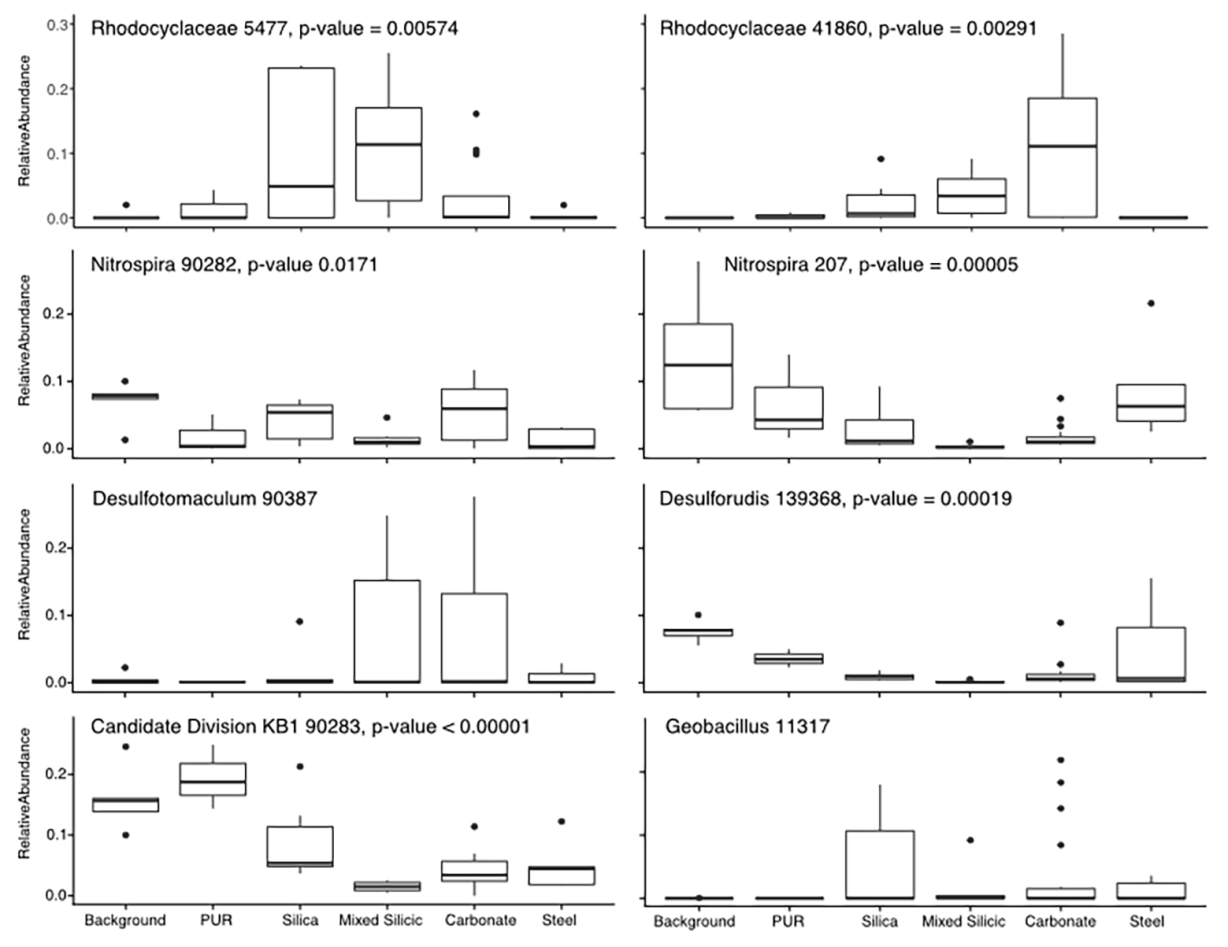
Tukey-style box and whiskers plot showing the average relative abundance of similarity percentage (SIMPER)-identified important taxa by substrate group. For each grouping of samples, the box represents the upper and lower quartiles surrounding the median (line in the box). Whiskers represent the remaining spread of the data, apart from outlying data points (defined as points further from the median than 1.5× Interquartile Range). We isolated 16S data from Incubations 1, 2, and 5 (since they are least impacted by the organic substrate addition) and identified the eight most common OTUs to appear in SIMPER pairwise analysis of substrates (ANOSIM by substrate: *R* = 0.3863, *p* = 0.001). The relative abundances for these OTUs were averaged across the three Incubations in order to analyze mineral preference across the microbial community. “Background” samples include bailed filtered and unfiltered water (*n* = 5); “PUR” indicates polyurethane foam block samples (*n* = 3); “Carbonates” include calcite, dolomite, and HVD samples (*n* = 18); “Silica” indicates glass wool samples (*n* = 6); “Mixed” indicates MS lithology (tuff and lake sediments; *n* = 6); “Steel” indicates low carbon steel ball bearings (*n* = 5). Plots are additionally labeled with the one-way ANOVA value of *p* if significant (*p* < 0.05). The lack of significance for the *Desulfotomaculum* and *Geobacillus* OTUs was indicative of the fact that the *Desulfotomaculum* population crashed after Incubation 1 and that the *Geobacillus* was infrequently found on minerals, but that, when present, it was often in high abundance, supporting the idea that this organism is infrequently detected but typically detected at high abundance.

To understand which OTUs were most affected by mineralogical variation, we focused on the subset of data least affected by the organic matter amendment (Incubations 1, 2, and 5). After confirming that the substrate bins were still a significant contributor to the variance in these three incubations (*R* = 0.3871, *p* = 0.001), we used pairwise SIMPER to identify OTUs contributing most toward the variance between substrate bins (Figure 3). The patterns in colonization of these OTUs indicate whether they were more commonly found in water samples or on one of the different lithological substrates. For example, the Candidate Division KB-1 OTU was the most abundant OTU recovered from water and PUR samples (17.3% relative abundance on average) and was moderately abundant in glass wool cartridges (8.9%), but consistently represented <5% of the community on mineral samples. Other OTUs that were enriched in planktonic samples included an *Nitrospiraceae* OTU (14.1%) and an OTU matching *Desulforudis* sp., a putative hydrogenotrophic, sulfate-reducing Firmicute discovered in deep strata accessed by a South African mine (7.7% abundance, 100% sequence ID; Moser et al., 2005; Chivian et al., 2008). Several OTUs which were abundant in water samples were also found on the low-carbon steel, including the OTU matching *Desulforudis* (5% average, 15.5% max abundance). Other OTUs appeared to show a preference for solid or mineral substrates. For example, an OTU identified as a *Geobacillus* sp. consistently represented 1–5% of the community on glass, steel, and mineral samples (up to 19% on a glass sample), but was only detected in 1 of 17 water or PUR samples at 0.08% relative abundance.

In general, analysis of relative abundance values can be difficult; values can change significantly depending on total community composition. It is important, therefore, to apply a transformation to these values to minimize the effect of small number variance (e.g., fourth-root transform, as we have done) and to contextualize relative abundance shifts by examining OTU overlap between different sample bins. Overall, across all incubations and samples, 75% of the OTUs identified on lithological substrates (carbonate, MS, silica glass wool, and steel) were detected at least once on each substrate (Supplementary Figure S6). Carbonate and MS shared the most OTUs unique to two categories (3.2%) and carbonate, MS, and silica glass wool shared the most unique to three categories (8.4%). Most of the variance, therefore, between substrate types was caused by differential abundance rather than strict OTU presence or absence.

We also used these techniques to attempt to identify which, if any, OTUs were correlated with the presence of the framboidal iron sulfides found on dolomite and HVD samples in Incubation 1. By subsetting our data to Incubations 1 and 2, which contained the same substrates and differed only in length of incubation, we attempted to identify microbial community shifts concurrent with iron sulfur framboid precipitation. Variance analysis (ANOSIM) confirmed that the community colonizing dolomite samples (HVD and dolomite standard) significantly differed between Incubations 1 and 2 (*R* = 0.8125, *p* = 0.04). A SIMPER analysis identified the top two taxa responsible for driving the difference between dolomite samples in Incubation 1 and 2 as *Sulfurovum* sp., an *Epsilonproteobacteria*, and *Desulfotomaculum* sp., a Firmicute, which were detected in Incubation 1 at high abundances but in Incubation 2 were either completely absent (*Sulfurovum*) or at much lower relative abundance (*Desulfotomaculum*). Cultured representatives of the two genera are sulfide-oxidizers and sulfate-reducers, respectively.

### Community Response to Organic Substrate Amendment

**Figure 4:**
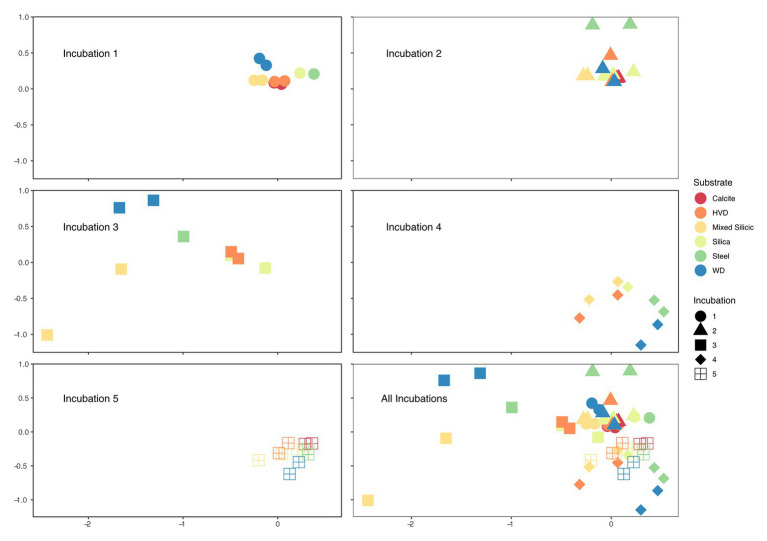
Faceted non-metric multidimensional scaling (NMDS) of microbial relative abundance on solid substrates used in each Incubation with combined NMDS bottom right. Each point on the plot represents an individual cartridge from an incubation; two points of the same color and shape are biological replicates of substrates from the same incubation but incubated in separated cartridges *in situ*. Incubations 1 and 2 are the most closely related and both occurred before the addition of the organic carbon substrate. Incubation 3 represents the first samples obtained after the carbon amendment and displays the widest variation amongst biological replicates and amongst mineral substrates. Incubation 4 is dominated by methanogenic archaea which pulls the samples into a cluster toward the bottom, and by Incubation 5, the samples are again tightly clustered and close to their starting position on the NMDS axes.

**Figure 5:**
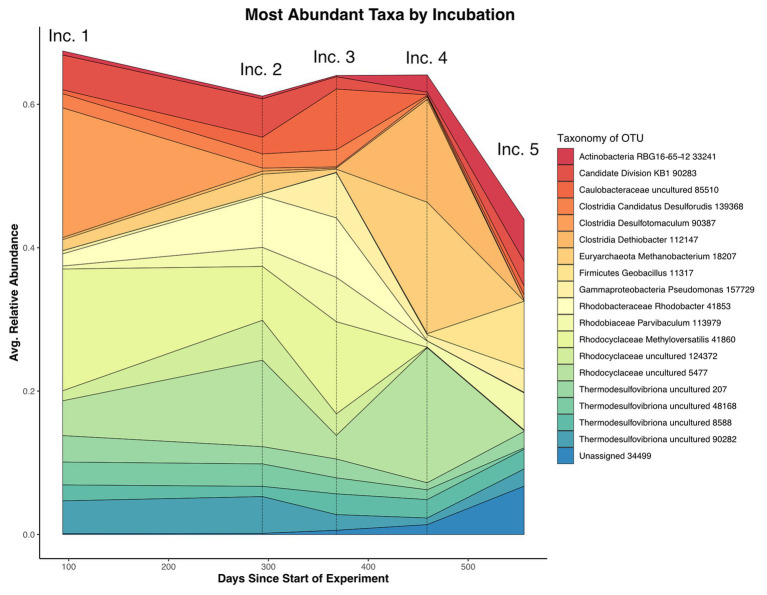
Average relative abundance of most abundant taxa by days since the start of the experiment, with individual incubation collection points noted above. The relative abundance as measured by 16S rRNA iTag data from mineral substrates common to all incubations was averaged at each time point. The data demonstrate the large relative abundance decrease in the *Desulfotomaculum* OTU co-occurent with the loss of framboidal iron sulfides in Incubation 2 as well as the large bloom in a *Methanobacter* OTU in Incubation 4 (6 months after organic substrate amendment). Incubation 5 displays a general increase in the proportion of rare taxa relative to the whole.

Upon retrieval of the Incubation 3 deployment, organic sponges from the 752 mbls region were missing, although the zip ties used to attach them were still on the line. We assume that the 752 mbls sponges were degraded during the incubation as a significantly degraded, gelatinous sponge was still attached to the line from the 579 mbls set. The close relationship between Incubations 1 and 2 and the dramatic community shift in Incubation 3 demonstrated the profound impact that the organic matter amendment had on the microbial community. Biological replicates of substrates in 4 of the 5 incubations (1, 2, 4, and 5) were closely clustered in the NMDS, but replicates from Incubation 3, which were incubated concurrent with the organic matter amendment, displayed the greatest separation from each other as well as from samples from the other incubations (Figure 4). Changes in relative abundance of the most abundant OTUs across all incubations are summarized in Figure 5.

The microbial assemblage colonizing the degraded sea sponges from the organic matter amendment experiment was distinct from that recovered on the mineral substrates during the same incubation. The top three OTUs recovered from the organic sponges are most closely related to *Fervidobacterium*, a Thermotogae (20.3% of recovered sequences), *Anaerolinea*, a Chloroflexi (19.0%), and *Thermodesulfovibrio*, a Nitrospiraceae (18.6%). These organisms dominated the sponge assemblage, but were only rare OTUs in samples from previous mineral incubations and water samples. We did observe these OTUs in the microbial assemblage on minerals adjacent to the organic sponge in Incubation 3 but at much lower relative abundance. For example, the next highest relative abundance of any of these sponge-dominating OTUs on any mineral substrate from this *in situ* deployment was an OTU related to *Anaerolinea* in the pyrrhotite cartridge (5.3% relative abundance). These findings indicate that a small subset of the microbial community was stimulated by spongin protein amendment but that shifts in the mineral-hosted community cannot be solely attributed to cells directly transferred from the organic matter-associated community. Mineral substrate assemblages from Incubation 3 displayed a relative increase in a few key taxa, including a putative carbon-degrading Rhodocyclales matching *Methyloversatilis* sp. (8.1–13.6%, 100% nucleotide identity) and an OTU identified as an uncultured *Caulobacteraceae* (2.3–8.5%), while the relative abundance of taxa commonly detected on minerals in previous incubations showed a marked decrease. These *Methyloversatilis* and *Caulobacteraceae* OTUs were undetected and 0.00013% of the community on the organic sponge, respectively, further suggesting that their presence on the neighboring mineral substrates in Incubation 3 was not simply due to cross-contamination from the organic sponge associated microbial assemblage.

The subsequent set of mineral cartridges in Incubation 4, recovered 6 months after the organic carbon amendment, had very few sequences of *Methyloversatilis* sp., with all but three cartridges (one dolomite, one HVD, and one MS) containing no detectable sequences from the *Methyloversatilis* OTU. Instead, a shift in community composition featuring methanogens was observed, with an OTU annotated as a *Methanobacter* sp. representing nearly 15% of the recovered diversity across all samples. The *Methanobacter* OTU, along with other archaea (*Methanothermobacter*, South African Gold Mine group Thermoplasmata), was detected in previous incubations but at much lower relative abundance (less than 2%; Figure 5).

The microbial assemblage recovered from Incubation 5, 9 months after the organic matter experiment, appeared to more closely resemble the original community composition in Incubations 1 and 2. Groups such as the Candidate Division KB-1, *Nitrospira* sp., and *Sulfurovum*, which decreased in their relative abundance during Incubations 3 and 4 likely due to a proportional increase in fast-growing microorganisms responding to organic matter amendment, returned to their original relative abundance in Incubation 5. Other OTUs became more prominent, and Incubation 5 samples had a comparatively higher diversity with higher Shannon indices (Supplementary Table S5). One OTU recovered from this incubation (4% average rel. abundance) matched sequences with no known phylum according to our cutoff (90% ID). This OTU had 99% similarity to a bacterial sequence recovered from hot springs (NCBI BLAST accession number KM740594.1), and was distantly related to Aminicenantia (87% ID) in the Silva database but was below our identity cutoff (National Library of Medicine, US, 1988). Other microbial lineages remained at low levels in Incubation 5, including members of the *Desulfotomaculum*, which was closely associated with the presence of iron-sulfide framboids in Incubation 1. The *Sulfurovum* OTU, which was also implicated in framboid formation, did recover to roughly the same relative abundance as in Incubation 1. Notably, the difference in *Desulfotomaculum* was identified in SIMPER analyses as the primary factor distinguishing communities in Incubation 1 and 5. Apart from these differences, however, the net result of these analyses indicated that much of the community composition within this borehole environment was re-established less than a year after organic matter perturbation.

## Discussion

### Comparison of Inyo-BLM 1 to Other Deep Biosphere Environments

Although the timescale for mineral substrate colonization in the deep biosphere is not well constrained, we infer that the substrate-attached microbial assemblages we detected in our experiments were representative of natural assemblages and likely not introduced contaminants. Prior to our experiments, the anoxic and 57°C Inyo-BLM 1 borehole sat undisturbed for 3 years, creating conditions that are generally unfavorable to the persistence of contaminating microbial organisms (Pedersen et al., 1997). A previous study in a deep South African mine borehole demonstrated that natural microbial assemblages and the *in situ* condition of anoxia returned within 2 months of perturbation (Moser et al., 2003). Additionally, the microorganisms observed in Inyo-BLM 1 were remarkably similar to 16S rRNA gene clone library observations originally reported in this South African gold mine (Moser et al., 2005). Conditions at this site were similar to Inyo-BLM 1, characterized by warm (54°C), anoxic, paleometeoric waters upwelling from 4 to 5 km depth into a metabasaltic unit underlying dolomite in South Africa. Clone libraries in that study detected four main taxa in the community: *Desulfotomaculum*, *Comamonadaceae* (Betaproteobacteria), *Candidatus* Desulforudis audaxviator, and *Methanobacterium*. Inyo-BLM 1 contains OTUs identified as all of these groups at high relative abundance, including *Methanobacterium* (100% match over length to Moser et al., 2005 sequence) as the dominant archaeon. Since their identification in 2008, the putative sulfate-reducing hydrogenotrophs *Desulforudis* have been reported from an array of deep biosphere environments (Chivian et al., 2008; Tiago and Verissimo, 2013; Jungbluth et al., 2017). Interestingly, many of these studies, including the original description, were performed in boreholes with steel casings, as is the case for Inyo-BLM 1. It is likely that steel casings, especially low-carbon steel, may be contributing to local H_2_ production that enriched these organisms. Beyond the taxa it shares with other deep biosphere studies, Inyo-BLM 1 contains a number of unusual bacterial lineages, including representatives from Candidate Divisions KB-1 and Aminicenantes (formerly OP8). Candidate Division Aminicenantes were initially described from the Obsidian Pool thermal spring in Yellowstone National Park and have since been detected in diverse marine and terrestrial subsurface environments (Hugenholtz et al., 1998). Although no cultured representatives are available, recent metagenomic analysis of Aminicenantes recovered from a Siberian deep thermal aquifer indicated a potential for complex carbohydrate and proteinaceous substrate fermentation as well as heterotrophic nitrite respiration (Kadnikov et al., 2019). Originally discovered in the Kebrit Deep brine basin in the Red Sea (Eder et al., 1999), Candidate Division KB-1 bacteria have since been identified in a number of other hypersaline environments, including other deep hypersaline brine lenses, salterns, and hypersaline lakes and sediments (Nigro et al., 2016). Today, the Inyo-BLM 1, and the LCA that feeds it, are low salinity, but the Amargosa Desert region was home to evaporitic, fluvial lakes in the Paleozoic (Khoury et al., 1982) and it is interesting to consider whether these as yet uncultured bacteria may have adapted over time to the freshening groundwater environment.

### Substrate Specificity of Microorganisms

The physical and chemical environment in the terrestrial deep biosphere is often extremely heterogeneous both in terms of the mineralogy as well as the nature of the permeating fluids. While fluid sampling is common in deep rock-hosted biosphere studies through legacy boreholes and mines, the ability to study mineral-associated microbial assemblages in the deep subsurface is more challenging (Moser et al., 2003; MacLean et al., 2007; Davidson et al., 2011; Ino et al., 2016). Previous work in the shallow subsurface has demonstrated that planktonic and mineral-attached communities are distinct, with some species significantly more abundant in one fraction or the other (Flynn et al., 2013). Here, we attempted to understand the distinction between the planktonic and mineral-attached communities as well as the role that mineral heterogeneity plays on microbial colonization by incubating a set of minerals representing the *in situ* lithologies at Inyo-BLM 1 alongside mineral standards and comparing the attached microbial assemblages to the planktonic community recovered from the aqueous phase. Similar types of mineral colonization experiments in both terrestrial and marine environments have pointed to local mineralogy as a primary factor linked to *in situ* microbial diversity (Roberts, 2004; Edwards et al., 2005; Orcutt et al., 2010; Toner et al., 2013). In some cases, microbial attachment is associated with direct mineral respiration, as occurs with iron-reducing bacteria (Lovley et al., 1989; Caccavo and Das, 2002), and in other circumstances, attachment may be controlled by the surface chemistry or electrostatic interactions between cell membranes and the mineral surface (Hermansson, 1999; Yee et al., 2000; Tuson and Weibel, 2013).

Nearly all of the OTUs we recovered from Inyo-BLM 1 were recovered from the solid substrates; 27.2% of the OTUs were exclusively detected on solid substrates, whereas only 0.4% were unique to the water samples (Supplementary Figure S6). Of the OTUs that were the most common contributors to substrate differentiation, a Candidate Division KB-1 OTU (bin90283) was abundant both in the water samples and on the PUR samples, and a *Geobacillus* OTU (bin11317) was only abundant on solid substrates (Figure 3). Notably, the abundance of *Geobacillus* was not significantly different as assessed by ANOVA, because many samples, even the solid substrates, had no detectable sequences from this lineage. Its variance on solid substrates, however, was much higher, with abundance on carbonate samples ranging from 0 to 21.9%. An F-test for significantly different variance confirmed that all of the solid substrates (carbonates, MS, silica, and steel) all had significantly higher variance than water samples for the relative abundance of this OTU, implying that the *Geobacillus* species only rarely colonized the minerals in the cartridges, but that when this occurred, these organisms frequently occurred at relatively high abundance.

Of the 518 OTUs found on the solid substrates, 72% of the OTUs were detected on least one sample from each lithological substrate, while only 5% of the OTUs were unique to a single lithological substrate type (Supplementary Figure S6). The mineralogy seems to play a significant role in governing the relative abundance of many OTUs and recruiting specific microbial lineages, both at the class and genus level, and differentiating among predicted microbial guilds (Figure 3). Twenty-nine OTUs of *Rhodocyclaceae*, a family of Betaproteobacteria, were identified in our survey, with very different colonization patterns. For example, a *Methyloversatilis*-affiliated OTU was more than twice as abundant on average on the carbonate minerals (calcite, dolomite, and HVD) than on glass wool or the MS sediments, while another *Rhodocyclaceae* OTU was most abundant on the glass wool and silica-rich MS. The underlying ecological or physiological factors influencing mineral colonization preference for these uncultured groups are not known, but the persistent partitioning by mineral substrate across the different incubations is notable. In another example, two OTUs affiliated with *Desulforudis* sp. (bin139368) and *Desulfotomaculum* sp. (bin90387), both putative sulfate-reducing Firmicutes, demonstrated different patterns of colonization – *Desulforudis* was most abundant on the low-carbon steel and *Desulfotomaculum* was most abundant on carbonate minerals. Notably, the ANOVA analysis of the *Desulfotomaculum* species across Incubations 1, 2, and 5 did not indicate that the difference was statistically significant, because that particular OTU was detected only at very low abundance after Incubation 1. Within Incubation 1, however, that *Desulfotomaculum* OTU averaged 20.9% of the recovered community from the carbonates vs. 1.4% of the community on the steel. Although inferring metabolic potential from 16S rRNA data represents a tentative assignment only, sequenced genomes *Desulforudis* indicate that they are capable of autotrophic growth through hydrogen-coupled sulfate reduction, and we hypothesize that *Desulforudis* in Inyo-BLM 1 may be locally consuming H_2_ produced by the anaerobic corrosion of the steel (Chivian et al., 2008; Jungbluth et al., 2017; Supplementary Figure S4). Sequenced genomes and cultivated representatives of *Desulfotomaculum* are typically heterotrophic sulfate-reducers (Aüllo et al., 2013; Sousa et al., 2018), and although the exact reason for their preferential association with carbonates in our data is unclear, the natural tendency of those minerals to entrain organic matter as they form may be one possibility (Roberts et al., 2013). A distinct colonization pattern was observed with yet another *Desulfotomaculum*-identified OTU (bin86994), which was most abundant on low-carbon steel, pyrite, and pyrrhotite mineral substrates. The intra-genus colonization differences between putative sulfate-reducing Gram-positive bacteria across different mineral substrates highlights the value of examining colonization patterns at the OTU level rather than higher-order taxonomic clustering.

The three carbonate species used in our flow-through cartridges (calcite, dolomite, and HVD) demonstrated overall similar communities and were not significantly different in the iTag sequencing data when comparing across all samples. These findings differ from some studies examining authigenic calcite, aragonite, and dolomite at deep-sea methane seeps, which did observe community variations between calcite and dolomite substrates (Case et al., 2015). SEM scans did reveal, however, that both Incubation 1 dolomite mineral samples (HVD and dolomite standard) were substantially modified *in situ* by the precipitation of framboidal iron sulfides, whereas calcite samples from the same incubation had no observable secondary mineral precipitation (Figure 2). It is possible that the difference in colonization patterns observed in marine studies was due to increased sensitivity of marine microorganisms to carbonate mineralogy because carbonate is a more dominant lithology in marine environments than terrestrial. The colonization differences between carbonate minerals may be more subtle than the differences between carbonate phases and other mineral families. Increasing the sample size of calcite and dolomite incubations in future deployments may help resolve whether there are discernable differences between the colonization of different carbonate minerals.

The specific mechanisms underpinning mineral preferences are currently unknown, but the repeatable patterns observed at the Inyo-BLM 1 site highlight potential ecological partitioning between subsurface taxa associated with minerals. Mineralogical groupings were a significant determinant of microbial community variance in most of our samples. Aside from Incubation 3, when complex organic matter was introduced, cartridges with duplicate lithologies acted as robust biological replicates, closely associated in the NMDS analysis (Figure 4) and ANOSIM R statistics for mineral type variation for were high (>0.35). In Incubation 1, nearly all of the variation between samples was attributable to mineral type (*R* = 0.9733). The Incubation number, partially representing the temporal distance from the organic carbon amendment, was a more important component when considering the entire data set, but perturbations on the scale of our experiment are likely infrequent. Since the vast majority of microorganisms in the deep biosphere are presumed to be mineral-attached (McMahon and Parnell, 2014), our experiments highlight the potential for targeting certain metabolisms and organisms based on the geology of their environment.

### Framboidal Iron Sulfide Precipitation

Framboidal iron sulfides, especially pyrite, have long been considered a potential biomarker, owing to the fact that they are often found in areas of high microbial activity, such as peat bogs and biofilms (Popa et al., 2004; Picard et al., 2016). It remains unknown, however, whether framboids are formed as a direct result of microbial activity or abiotically due to geochemical conditions (Sweeney and Kaplan, 1973; Graham and Ohmoto, 1994). As MacLean noted (MacLean et al., 2008), however, laboratory abiotic syntheses have only been accomplished at high-temperatures (above 60°C), well above the temperatures at nutrient- and biomass-rich systems where framboids are frequently found (Sawlowicz, 2000; Popa et al., 2004). It has been demonstrated that organic matter may play a role in aggregating the individual crystals into the framboid formation (MacLean et al., 2007) and MacLean et al. (2008) observed individual crystals developing within an organic matrix. Particularly interesting in our study was the correlation between the presence of secondary iron sulfide framboids on two independent dolomite incubations and two OTUs corresponding to *Desulfotomaculum*, a presumed sulfate-reducer, and *Sulfurovum*, a putative sulfide-oxidizing bacterium. Mineralogical heterogeneity of the substrate did not appear to explain the observed variation. Although both dolomites contained potential mineralogical sources for the framboids (higher iron content in the dolomite standard and micron-scale, anhedral iron- and sulfur-rich regions in the HVD), the secondary framboids formation was only observed during Incubation 1, although the same minerals were used for the incubations throughout the 18-month experimental series. Precipitation of iron sulfides also could not be attributed to incubation time, as under optimal conditions, pyrite framboids have been shown to form within days (Rickard, 2019). Even if the organic matter amendment during Incubation 3 disrupted the ecological conditions, therefore, we would have expected to observe framboid formation in the Incubation 2 (197 days long). While sulfide concentrations were below detection in the water recovered from the borehole, it is possible that localized sulfur metabolism by *Desulfotomaculum* and/or *Sulfurovum* directly influenced framboid precipitation. The groundwater feeding Inyo-BLM 1 does contain trace iron and zinc (1.4 and 0.06 μM, respectively; Supplementary Table S2), which can react with sulfide and may contribute to undetectable aqueous sulfide concentrations in our water samples.

In addition to their ability to respire sulfate, it has been shown that some *Desulfotomaculum* are capable of growth as fermentative syntrophs coupled with hydrogenotrophic methanogens, rather than heterotrophic sulfate reducers (Imachi et al., 2000, 2006; Plugge et al., 2002; Moser et al., 2005). Based on our 16S rRNA data alone, it is difficult to assess the physiology of these taxa in Inyo-BLM 1. On one hand, the detection of hydrogenotrophic methanogens affiliated with *Methanobacterium* in Inyo-BLM 1, the common partner of *Desulfotomaculum* when growing syntrophically (Imachi et al., 2000; Moser et al., 2005), suggests a similar syntrophic interaction could occur in our system; however, the co-occurrence of *Desulfotomaculum* (bin90387) with iron sulfides also supports a possible role in sulfur metabolism. Our laboratory experiments with steel alloys confirmed that the steel we added in each of the *in situ* incubations could have resulted in elevated H_2_ *via* anaerobic corrosion (the Schikorr reaction, e.g., $Fe^{0}+2H_{2}O\to Fe^{2+}+2OH^{-}+H_{2}$; Schikorr, 1929; Linnenbom, 1958; Ma et al., 2013). Therefore, we cannot dismiss the possibility that these organisms are syntrophs, that their disruption following Incubation 1 was due to inhibitory concentrations of hydrogen, and that their correlation with pyrite formation was related to their role in producing small organic carbon molecules which aid in aggregating iron sulfide framboids rather than the production of sulfide.

### Organic Nutrient Amendment

In most environments, oxidation of organic matter proceeds by the sequential reduction of O_2_, NO_3_^−^, Fe^3+^, SO_4_^2−^, and CO_2_, in order of decreasing electron potential (Ponnamperuma, 1972). Because organic matter is thought to be move extremely slowly through the deep biosphere, most of these electron acceptors are long depleted at depth (Canfield, 1994; Hedges and Keil, 1995; Arndt et al., 2013; Osburn et al., 2014; Bradley et al., 2020). This results in a typically reduced system with low energy availability to the inhabiting organisms; Inyo-BLM 1 both has low dissolved organic carbon levels (0.431 ppm DOC) and few available electron acceptors (No detectable nitrate or nitrite and <2 mM sulfate; Supplementary Table S2). Deep biosphere microorganisms are therefore thought to have low energy requirements (LaRowe and Amend, 2015) and grow extremely slowly, with estimated turnover rates varying orders of magnitude from months to thousands of years (Morono et al., 2011; Xie et al., 2013; Onstott et al., 2014; Trembath-Reichert et al., 2017). Because of the long growth times and slow metabolic rates, ecological principles such as succession, competition, or dispersal, are likely reduced in impact and difficult to constrain (Biddle et al., 2011). By taking advantage of the addition of organic sponges to our system, we were able to observe the community response and some of these ecological forces at work in an unusual system. The microorganisms colonizing the sponges themselves were significantly different from the mineral-associated community, and in fact, the microbial diversity recovered from the incubated sponge mostly did not include OTUs that were abundant on the minerals, suggesting that the changes in the mineral-hosted community were driven by downstream degradation products of the sponges, analogous to the detrital matter that the deep biosphere likely subsists upon. The results imply that the planktonic Inyo-BLM 1 assemblage is a reservoir for microorganisms that are capable of colonizing and rapidly metabolizing complex organic matter. The mineral-colonizing community, by contrast, may be taking advantage of the small organic molecules released during the breakdown of the proteinaceous sponge biomass.

This organic matter amendment experiment also provides an opportunity to estimate community-wide carbon oxidation rates. The natural sponges introduced during Incubation 3 provided ~0.9 g of proteinaceous organic carbon and 0.3 g of fixed nitrogen. While tracing the organic degradation process and associated end-product production in this open system was not possible, we used the shifts in the microbial community as a general indicator of the ecological conditions after the organic matter addition. In our rough model, we assume during Incubation 3, when the sponges are first added to the well, that the microbial community is exposed to the most labile organic carbon species. A dramatic enrichment in *Methyloversatilis* sp. on the mineral substrates (Figure 3), but not on the sponge itself, indicates that this group perhaps is capable of rapid colonization and metabolism of organic molecules released by the sponge degradation. Cultured members of *Methyloversatilis* are methylotrophs that can oxidize a wide variety of C1 compounds coupled with oxygen or nitrate respiration (Lu et al., 2012; Doronina et al., 2014) and have been recovered in high abundances from oil reservoirs and hot springs (Lenchi et al., 2013; Doronina et al., 2014). The sudden dominance of these organisms in Incubation 3 and decrease in their relative abundance in subsequent incubations (Figure 5) suggests that they may represent a subset of the community poised to take advantage of carbon spikes to the system with a boom-bust lifestyle.

To our knowledge, this dolomitic aquifer is the first time organisms from Candidate Division KB-1 have been found in abundance in a terrestrial, low-salinity system. KB-1 is now classified as a subgroup of phylum Acetothermia (formerly OP1), which has been detected in anaerobic digesters as well as hot springs and other deep biosphere studies (Takami et al., 2012; Zaitseva et al., 2017; Hao et al., 2018), although the recovered diversity of related organisms in our system fall within the more narrowly defined KB-1 clade (sometimes referred to as class Acetothermiia) and we have therefore retained that name here. The only sequenced and published genome for Candidate Division KB-1, recovered from the Red Sea, points to a central metabolism based on glycine betaine degradation, a common N-containing organic osmolyte in hypersaline environments but less likely to be found in abundance in non-marine environments (Nigro et al., 2016). Although it is difficult to predict the metabolism of the as-yet uncultured KB-1 organisms in the groundwater due to the lack of extensive genomic characterization of this clade, the dynamics of community changes during the *in situ* organic matter addition, where the KB-1 OTUs dropped to very low abundance values over Incubations 3 and 4, suggest they were not stimulated (Figure 5). There are a number of possibilities for the observed decrease in KB-1 relative abundance, including that the KB-1 strains in this environment do not metabolize amino acids or that they perhaps were outcompeted by other microbes following the perturbation. Acetothermia and KB-1 organisms detected in other studies have revealed that the clade is capable of thriving in a range of organic matter concentrations, but if the KB-1 organisms in Inyo-BLM 1 are indeed heterotrophic (as are all the physiologically described representatives of this clade), our results imply that they are likely better adapted to *in situ* organic levels than to the excess provided during our amendment (Nigro et al., 2016; Zaitseva et al., 2017; Hao et al., 2018).

The recovery of Incubation 4 occurred 6 months after the introduction of the sponges to the well, and at this time, the microbial assemblage appeared to transition to a methanogenic phase. A small number of methanogen-affiliated sequences were detected in Incubation 1, including *Methanobacterium*, *Methanothermobacter*, and *Methanolobus*; however, samples from Incubation 4 were dominated by the OTU affiliated with *Methanobacterium*. All characterized isolates belonging to the *Methanobacterium* genus are hydrogenotrophic or formate-utilizing methanogens. Unexpectedly, although many OTUs associated with syntrophic clades (e.g., *Syntrophomonadaceae*, *Syntrophobacterales*) were observed during the organic enrichment in Incubation 3, a few were recovered from Incubation 4, suggesting that much of the complex fermentable carbon was consumed. By the time Incubation 5 was recovered (9 months after organic matter addition), the diversity of organisms colonizing the experimental substrates was largely consistent to the community composition observed during Incubations 1 and 2 prior to organic matter amendment, suggesting that the community had reset to the state prior to the perturbation (Figure 4).

The timing of the community shifts can provide bounds for order-of-magnitude activity estimates of the whole community in the borehole. Estimates for total carbon added to the system *via* diffusion were calculated above: 0.160–3.01 mmol. The lower activity estimate used the lower estimate for carbon diffusion (0.160 mmol C) and assumed that the methanogens detected were primarily using formate. Thus, the total estimated time for carbon oxidation to CO_2_ is 177 days, extending from midway through Incubation 3 through midway through Incubation 5 (the midpoints of the incubations were used approximately to allow time for methanogen abundance to decrease). Based on a cell count of 4.1 × 10^6^ cells/ml from Incubation 3, each cell is estimated to oxidize 0.02 fmol C per day on average. To calculate the upper bound on activity, we used the higher diffused carbon value (3.01 mmol) and instead assumed that the detected methanogen, *Methanobacterium*, is hydrogenotrophic, thus assuming that the maximum value for methanogen relative abundance is a proxy for the maximum in hydrogen concentration the exhaustion of reduced carbon species. Based on these assumptions, we estimate that the complete oxidation of the carbon amendment occurred halfway between the recovery of Incubation 3 and 4, which translates to a total oxidation time of approximately 120 days and an average activity rate of 0.577 fmol C per cell per day. This exercise serves as a rough order-of-magnitude estimation and predicts oxidation rates that are high, but within the margin of error as compared to carbon oxidation rates measured from other deep biosphere systems using more direct methods (0.002–0.0067 fmol C per cell per day; Morono et al., 2011; Trembath-Reichert et al., 2017; Moreno-Ulloa et al., 2019).

## Conclusion

The terrestrial deep biosphere is characterized by its phylogenetic, physiological, and geological heterogeneity. Mineralogical changes occur both regionally, as in lithological units, and at the microscale, with secondary mineral formation such as iron sulfides, potentially a product of the local micro-environment. This heterogeneity, along with the massive volume of the subsurface biome and difficulty in obtaining samples has meant that it is difficult to generate predictions as to the microbial population size, structure, or activity (Magnabosco et al., 2018). Although efforts are ongoing to address these questions, experiments performed in the laboratory are generally unable to capture all of the complexity of the subsurface. Our experiments serve as a wide-ranging *in situ* exploration of the factors controlling microbial populations and microbial colonization in one deep, warm dolomitic aquifer and enabled us to distinguish between populations of microorganisms living planktonically and those that preferentially adhere to a mineral surface. These observations provide some preliminary insight into the niche partitioning that occurs within the deep biosphere and emphasize the need for more comprehensive sampling of subsurface ecosystems. In addition to examining mineral-microbe associations, we utilized a complex N-rich organic matter amendment *in situ* to explore the response of the deep biosphere community to stimulation at this location. By observing this environment over a period of 9 months after the carbon amendment, we observed this community oxidize the organic carbon in several phases, with putative heterotrophic organisms closest to the carbon source, putative syntrophic organisms on neighboring mineral surfaces 3 months after the organic carbon were first deployed, and the dominance of methanogenic organisms 6 months after the original amendment. After 9 months, the community returned to the assemblage that closely resembled its undisturbed state. Our experiments indicate that the microbial community is highly responsive to shifts in geochemistry and nutrient availability; tracking these community changes can even provide coarse estimates for the activity of these microorganisms. More precise activity measurements could be obtained, perhaps utilizing stable isotopic tracers, but avoidance of contamination of the slow-moving environmental system may require any such measurements be *ex situ*. In any case, careful consideration of the activity of deep biosphere organisms will aid in our understanding of the dynamics of this massive and understudied system.

## Data Availability Statement

The datasets generated for this study can be found in the NCBI SRA # PRJNA605066.

## Author Contributions

SM coordinated field experiment set-up and sampling and performed DNA sequencing, sequence analysis, microscopy, and gas chromatography. GW coordinated field sampling, including the design of the *in situ* suspension line and provided microscopy data. BK, JS, and SH-B performed field sampling. Additionally, BK and JS coordinated retrieval and processing of geochemistry analyses. SH-B performed phase contrast cell counts. DM supervised BK, JS, and SH-B, identified and secured the field site, co-wrote the funding proposal, and organized downhole logging. RB, JA, and VO co-wrote the proposal and supervised field activities. VO helped conceive the experimental design and supervised SM. All authors contributed to the article and approved the submitted version.

### Conflict of Interest

Two authors declared commercial interests at the time of publication but not during the period of data collection: GW is employed by Oberland Agriscience, Inc., and RB is employed by Photon Systems, Inc.

The remaining authors declare that the research was conducted in the absence of any commercial or financial relationships that could be construed as a potential conflict of interest.
