# Supplementary material for: Patterns of in situ Mineral Colonization by Microorganisms in a ~60°C Deep Continental Subsurface Aquifer
Source: Front Microbiol. 2020 Nov 19;11:536535. doi: 10.3389/fmicb.2020.536535 (PMC7711152; doi:10.3389/fmicb.2020.536535)
Supplement: Supplementary file 1 [file Data_Sheet_1.docx]

**Supplemental Figures**

**Table S1**: Artificial BLM-1 media mimicking geochemistry at 579 m (in mg/L unless otherwise noted) :

| CaCl2*2H2O | 9.9 |
| --- | --- |
| MgCl2 | 25.1 |
| KCl | 30.5 |
| NaCl | 87.1 |
| NaHCO3 | 582.5 |
| KH2PO4 | 0.3 |
| Na2SO4 | 65.6 |
| SL-10 Trace Metal solution | 1 mL |
| KOH added until pH 9 |  |

**Table S2**: Geochemistry: Ions, trace ions, and dissolved gases of BLM-1 aquifer water, sampled 8/21/15 – 8/22/15

|  | **579 m** | **754 m** | **%Error** | Analysis Method (EPA Reference) | Dilution | MDL (mg/L) |
| --- | --- | --- | --- | --- | --- | --- |
| Al | 0.03 | 0.008 | 1 | M200.8.ICP-MS | 1 | 0.001 |
| Sb | 0.0008 | 0.0004 | 4 | M200.8.ICP-MS | 1 | 0.0004 |
| As | 0.19 | 0.0778 | 1 | M200.8.ICP-MS | 100,1 | 0.0002 |
| Ba | 0.018 | 0.1096 | 2 | M200.8.ICP-MS | 1 | 0.0005 |
| Be | U | U | 2 | M200.8.ICP-MS | 100 | 0.005 |
| B | 1.82 | 1.74 | 0 | M200.8.ICP-MS | 100 | 0.05 |
| Cd | U | 0.0002 | 4 | M200.8.ICP-MS | 1 | 0.0001 |
| Ca | 2.7 | 31.7 | 2 | M200.7.ICP | 1 | 0.1 |
| Cs | 0.0003 | 0.0037 | 4 | M200.8.ICP-MS | 1 | 0.0002 |
| Cr | U | U | 3 | M200.8.ICP-MS | 1 | 0.0005 |
| Co | U | 0.00016 | 1 | M200.8.ICP-MS | 1 | 0.00005 |
| Cu | U | 0.0006 | 1 | M200.8.ICP-MS | 1 | 0.0005 |
| Fe | 0.05 | 0.08 | 3 | M200.7.ICP | 1 | 0.02 |
| Pb | 0.0002 | 0.0006 | 2 | M200.8.ICP-MS | 1 | 0.0001 |
| Mg | 3 | 12.1 | 2 | M200.8.ICP-MS | 1 | 0.2 |
| Mn | 0.002 | 0.0958 | 1 | M200.8.ICP-MS | 1 | 0.0005 |
| Mo | 0.1201 | 0.0533 | 4 | M200.8.ICP-MS | 1 | 0.0005 |
| Ni | 0.002 | 0.0092 | 2 | M200.8.ICP-MS | 1 | 0.0006 |
| K | 16.1 | 17 | 2 | M200.7.ICP | 1 | 0.2 |
| Se | 0.0005 | U | 4 | M200.8.ICP-MS | 1 | 0.0001 |
| Si | 10.6 | 43.8 | 3 | M200.7.ICP | 1 | 0.2 |
| Ag | U | U | 9 | M200.8.ICP-MS | 1 | 0.00005 |
| Na | 215 | 222 | 3 | M200.7.ICP | 1 | 0.2 |
| Te | U | U | 5 | M200.8.ICP-MS | 1 | 0.001 |
| Tl | U | U | 5 | M200.8.ICP-MS | 1 | 0.0001 |
| Th | U | U | 3 | M200.8.ICP-MS | 1 | 0.001 |
| Sn | U | U | 2 | M200.8.ICP-MS | 20 | 0.002 |
| U | 0.0002 | 0.0002 | 3 | M200.8.ICP-MS | 1 | 0.0001 |
| V | U | U | 2 | M200.8.ICP-MS | 1 | 0.0002 |
| Zn | 0.049 | 0.004 | 1 | M200.8.ICP-MS | 1 | 0.002 |
|  |  |  |  |  |  |  |
| HCO_3_^-^ | 328 | 437 | 13 | SMB2320B- Titration | 1 | 2 |
| CO_3_^2-^ | 88.1 | U | 13 | SMB2320B- Titration | 1 | 2 |
| Hydroxide | U | U | 13 | SMB2320B- Titration | 1 | 2 |
| Total Alkalinity | 416 | 437 | 13 | SMB2320B- Titration | 1 | 2 |
| Hardness | 19 | 129 | 13 | SM2340B-calc | NA | 0.8 |
| TOC | 1.64 | 4.21 | 16 | SM5310B | 1 | 0.1 |
| DOC | 0.813 | 0.431 | 2 | SM5310B | 1 | 0.1 |
| POC | 0.83 | 3.78 | 17 | calculation from TOC-DOC | 1 | 0.1 |
| NO_3_^-^ | 0.02 | U | 1 | calculation from total nitrate/nitrate and nitrite | 1 | 0.02 |
| NO_3_^-^/NO_2_^-^ | 0.02 | U | 1 | M353.2 - Automated Cadmium Reduction | 1 | 0.02 |
| NO_2_^-^ | U | U | 0 | M353.2 - Automated Cadmium Reduction | 1 | 0.01 |
| NH_3_ | U | 0.22 | 0 | M350.1 | 1 | 0.05 |
| PO_4_^3-^ | 0.19 | 0.12 | 31 | Calculation based on dissolved orthophosphate | 1 | 0.03 |
| Phosphorus, ortho, dissolved | 0.06 | 0.04 | 31 | M365.1 Automated Ascorbic Acid | 1 | 0.01 |
| SO_4_^2-^ | 44.4 | 158 | 1 | D516-02/07 Turbidimetric | 5 | 5 |
| Cl^-^ | 49.2 | 52.4 | 8 | SM4500Cl-E | 1 | 0.5 |
| F^-^ | 3.93 | 3.98 | 15 | SM4500F-C | 1 | 0.05 |
|  |  |  |  |  |  |  |
| H_2_ | 0.02 | U | 21 | Described in main text | | |
| N_2_ | U | U | NA |  |  |  |
| CO_2_ | U | 0.22 | 22 |  |  |  |
| CH_4_ | 0.19 | 0.12 | 36 |  |  |  |
| CO | 0.06 | 0.04 | 10 |  |  |  |
| O_2_ | 44.4 | 158 | 62 |  |  |  |

**Table S2:** Major ions and nutrients found in the groundwater within the borehole were measured at two depths, one inside the casing (579 m) and one below the casing (754 m). The ground water samples were obtained by successive sampling with 3 and 1 L discrete sampling devices (respectively) on 8/22/15-8/23/15, before the organic matter amendment was added to the system on 8/23/15. “U” indicates measured values below the minimum detection limit (MDL). The Environmental Protection Agency (EPA) or National Environmental Methods Index (NEMI) method reference number is listed, and the percent error of detection method was also quantified by technical duplicates. Because of the inherent error introduced by sampling from a dissolved gas bottle, percent error is significantly higher in our dissolved gas measurements. The microbial community contains no indication that the system is aerobic at any point, and is dominated primarily by putative fermentative organisms and methanogens. We therefore consider the dissolved oxygen data as a sampling artefact and indicative of amount of leak in the gas sampling process.

**Fig S1:** NMDS of blank substrates vs Inc. 1


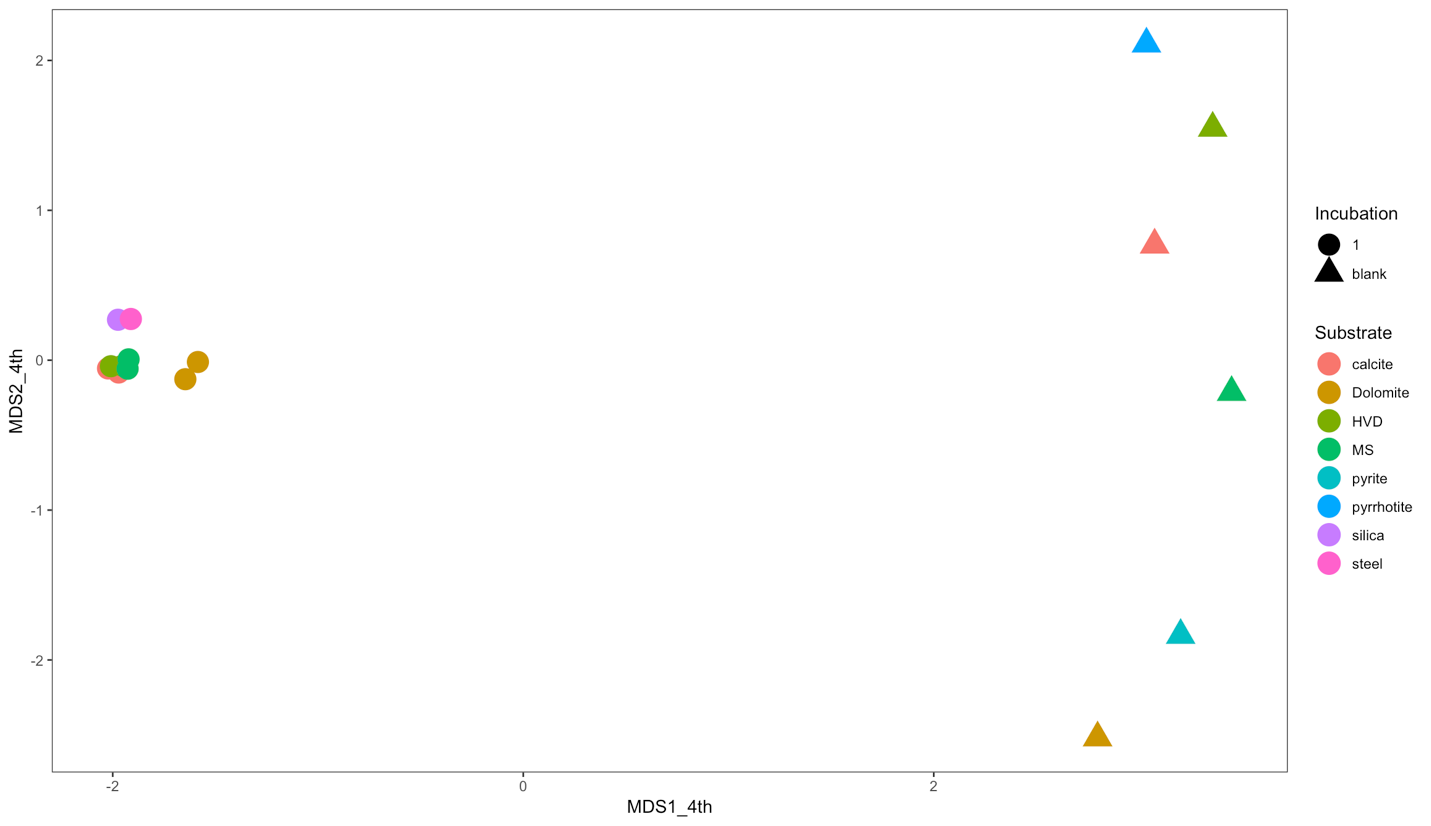


**Fig. S1**: All Incubation substrates were autoclaved before being incubated in the borehole. 16S rRNA genes did not amplify from these samples under standard conditions, but we were able to force a band to appear by increasing total PCR cycles to 45. Those amplified genes were also analyzed via Illumina iTAG. The NMDS shown is for all amplicons, including contaminants identified by a no-template PCR control, which makes these points even closer than they would be after contaminant removal. ANOSIM analysis indicates that >95% of the variance is explained by the “Incubation number” (R value = 0.9689, p value = 0.001)


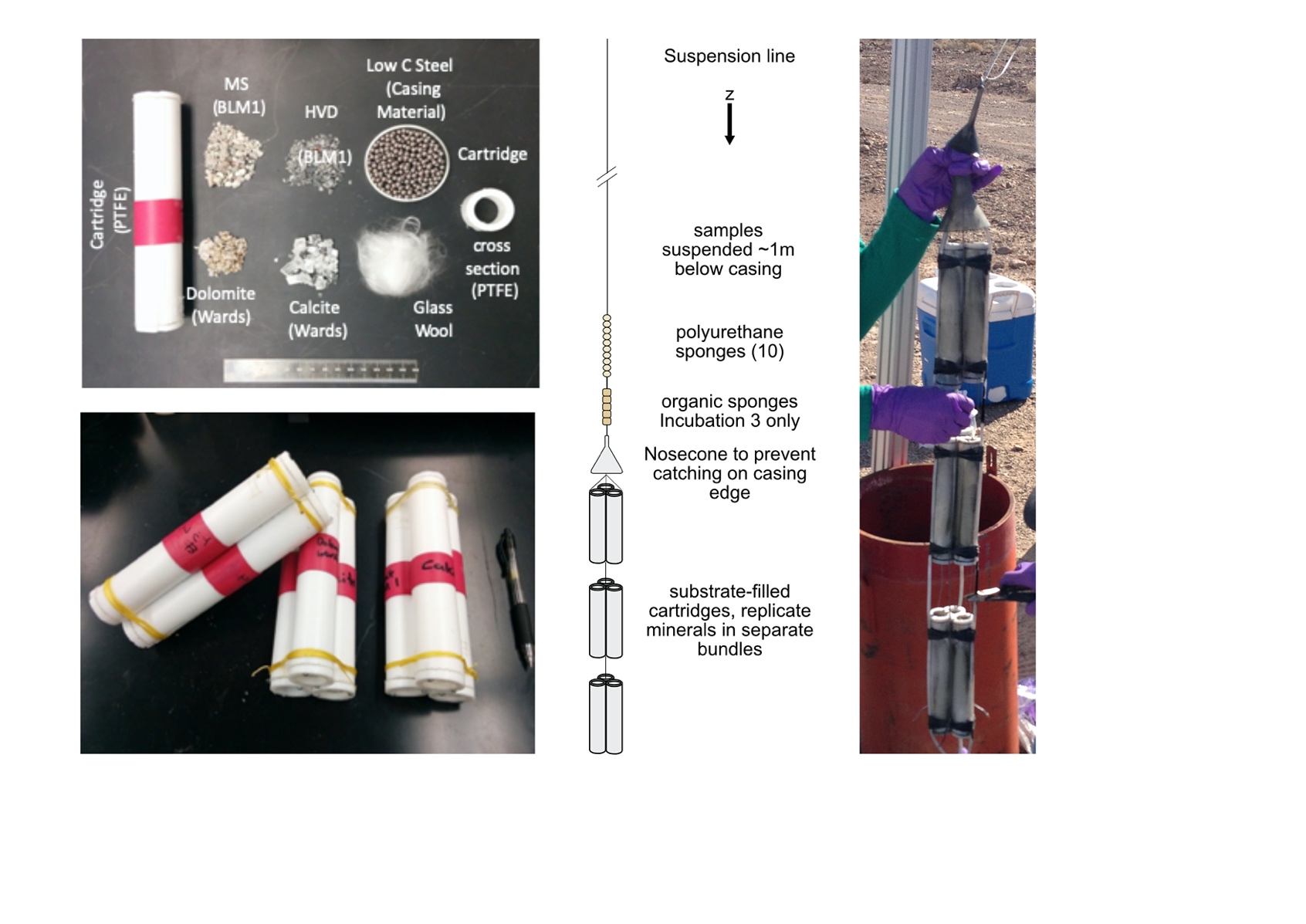


**Fig S2:** (Left) Images of PTFE cartridges with mineral substrates. Cartridges were filled with material, wrapped in bundles with high-test line, then autoclaved and kept sterile until their deployment. (Center) Schematic of bundles and sponge deployment in the borehole. (Right) Image of post-incubation retrieval of cartridge bundle. The borehole cap in orange and part of the aluminum suspension frame are visible in the background.

**Fig S3:** NMDS of all samples


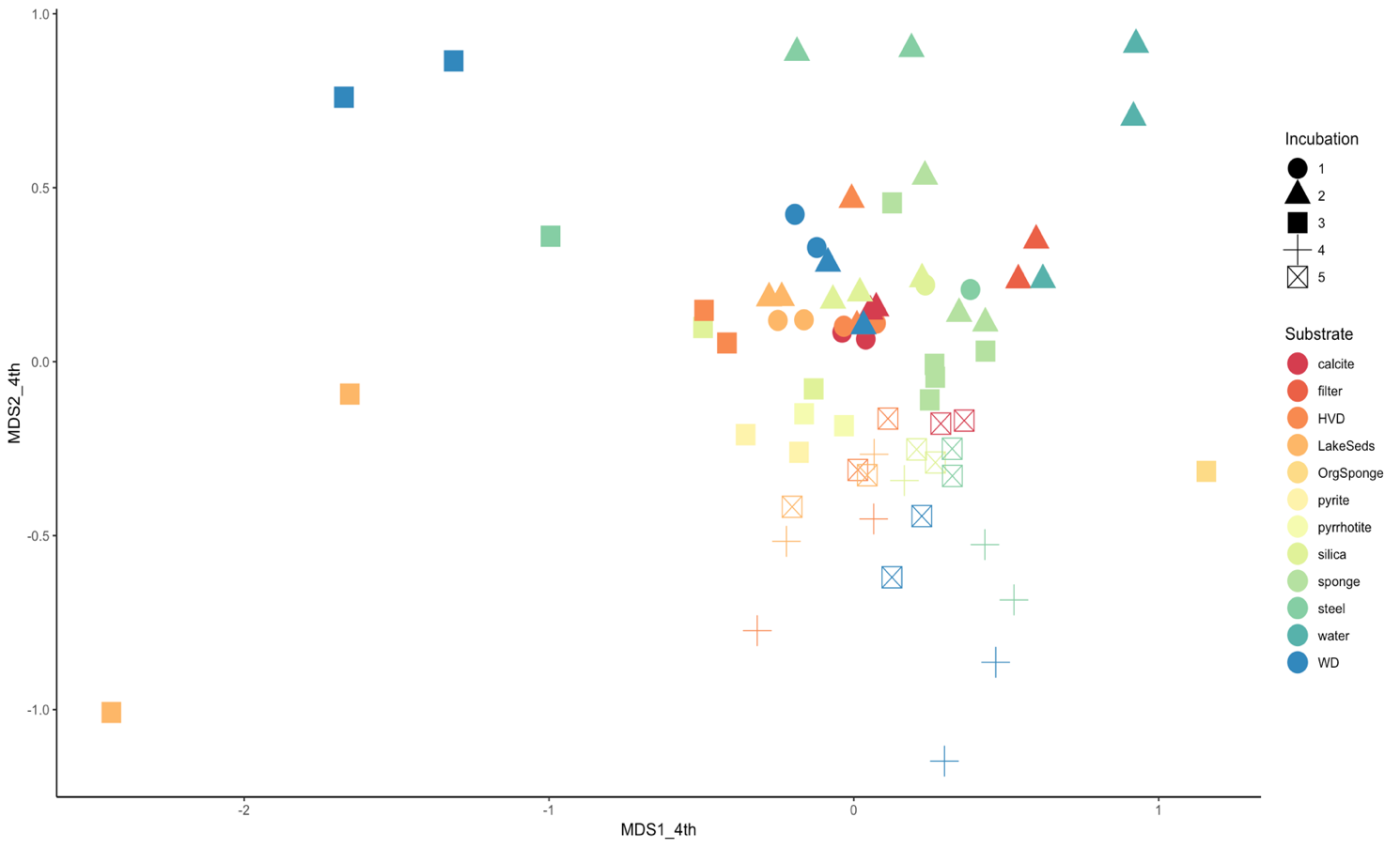


**Fig. S3:** NMDS of all samples taken from BLM-1, including raw and filtered water, minerals, polyurethane sponges, and the single recovered organic sponge. ANOSIM statistics for the complete dataset are as follows for the following variables: Incubation Number (0.4535, p = 0.001), specific substrate (0.2667, p = 0.001), substrate bin (0.3235, p = 0.001). There are three bins of substrates: carbonate (calcite, dolomite, HVD), sulfide mineral (pyrite, pyrrhotite), and planktonic (raw water, filtered water, polyurethane sponges); other substrates are treated as their own bin.


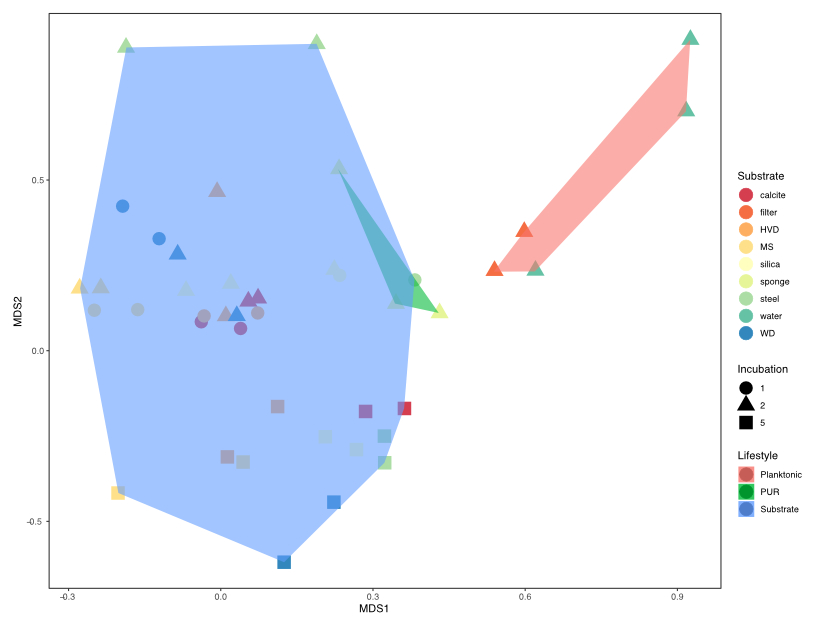


**Fig. S4:** NMDS of substrates for Incubations 1, 2, and 5, with hulls drawn around planktonic versus substrate groupings. The statistical significance of the lifestyle designations (planktonic, PUR, or substrate-attached) was assessed by ANOSIM (R = 0.4672, p-value = 0.001)

**Table S3:** Cell Counts at 2 depths. Cells were counted on August 21^st^, 2015 using a Petroff-Hausser counting chamber, using 10 "samplings" of counted areas for each individual water sample from Inyo-BLM1.

| Sample 8-20-2015 579 m | | | |  |
| --- | --- | --- | --- | --- |
| Sample # | Average cell count per square | Cells/mL | Average (cells/mL) | Std Dev |
| #1 | 0.15 | 3.0E+06 | 4.0E+06 | 1.0E+06 |
| #2 | 0.25 | 5.0E+06 |  |  |
| #3 | 0.2 | 4.0E+06 |  |  |
|  |  |  |  |  |
| Sample 8-21-2015-750 m | | | |  |
| Sample # | Average cell count per square | Cells/mL | Average (cells/mL) | Std Dev |
| #1 | 0.09375 | 1.9E+06 | 4.1E+06 | 2.8E+06 |
| #2 | 0.156 | 3.1E+06 |  |  |
| #3 | 0.36 | 7.2E+06 |  |  |

**Table S4:** X-Ray Fluorescence (XRF) mineral composition data. The four primary minerals including the calcite and dolomite standards and HVD and MS natural samples were analyzed for their elemental composition. Although the data are reported as weight percentages of the mineral oxides, XRF cannot detect light elements (including carbon or oxygen) or the redox state of elements. These values are therefore useful as a comparative tool, but are not an absolute measurement of composition or mineralogy.

|  | **Weight percentage** | | | |
| --- | --- | --- | --- | --- |
| **Oxide** | **Calcite** | **Dolomite** | **Hidden Valley Dolomite (HVD)** | **Mixed Siliclastic (MS)** |
| CaO | 99.781 | 71.238 | 70.187 | 17.291 |
| MgO | ⏤ | 25.259 | 25.818 | 4.103 |
| SiO_2_ | ⏤ | 1.544 | 3.295 | 58.151 |
| Al_2_O_3_ | ⏤ | ⏤ | ⏤ | 11.116 |
| Fe_2_O_3_ | 0.000356 | 1.576 | 0.53 | 3.054 |
| K_2_O | ⏤ | ⏤ | ⏤ | 5.631 |
| Ni_2_O_3_ | 0.00012 | ⏤ | ⏤ | 0.000013 |
| MnO_2_ | 0.058 | 0.343 | 0.138 | 0.106 |
| TiO_2_ | ⏤ | ⏤ | ⏤ | 0.418 |
| CuO | 0.00018 | ⏤ | ⏤ | 0.00002 |
| ZnO | ⏤ | 0.000115 | 0.000067 | 0.000087 |
| SrO | 0.112 | 0.000263 | 0.00021 | 0.0005 |
| PbO | ⏤ | ⏤ | 0.00021 | ⏤ |
| Y_2_O_3_ | ⏤ | 0.000029 | ⏤ | 0.000026 |
| **Total** | **99.951656** | **99.960407** | **99.968487** | **99.870646** |

**Fig. S5**: H_2_ Production from different iron sources. N=2 for each iron source: low carbon steel, 304 stainless steel, 316 stainless steel or crushed hematite. For each tube, three 3mm ball bearings of steel alloy or 0.5g crushed hematite were incubated anaerobically, in the dark, at room temperature in 10 mL defined sterile media, with pH and salt concentrations matching those of the 579 m water sample taken from the borehole. Each tube was sampled destructively, that is, we did not perform repeat measurements from individual tubes.

**Fig. S6: Venn’s Diagrams of OTU overlap on substrates**

**
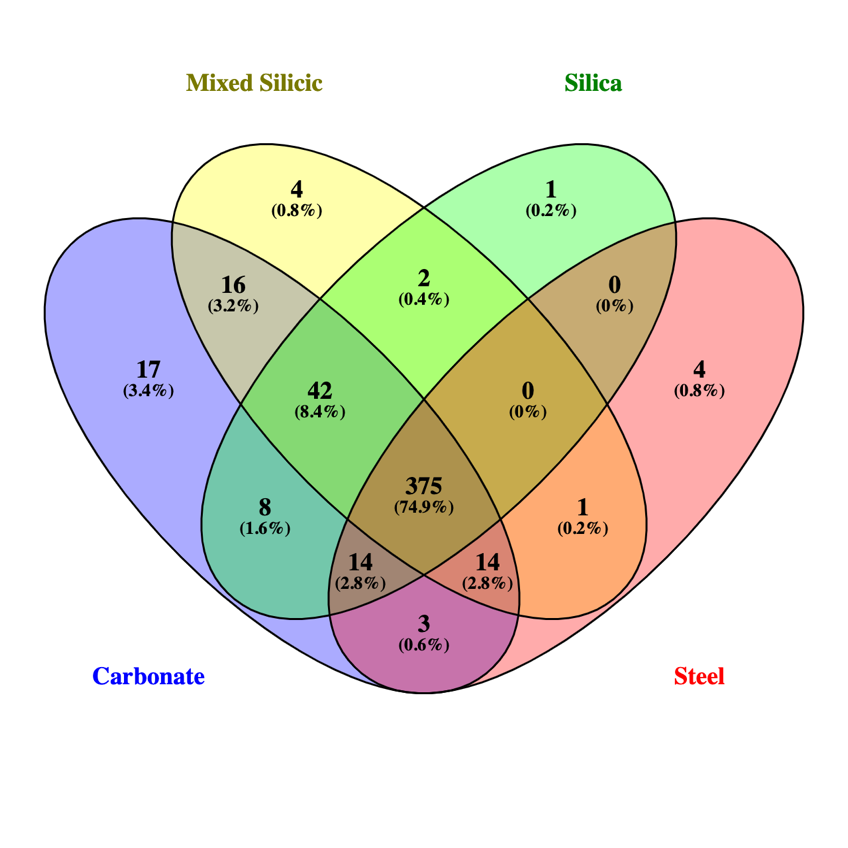
**

**
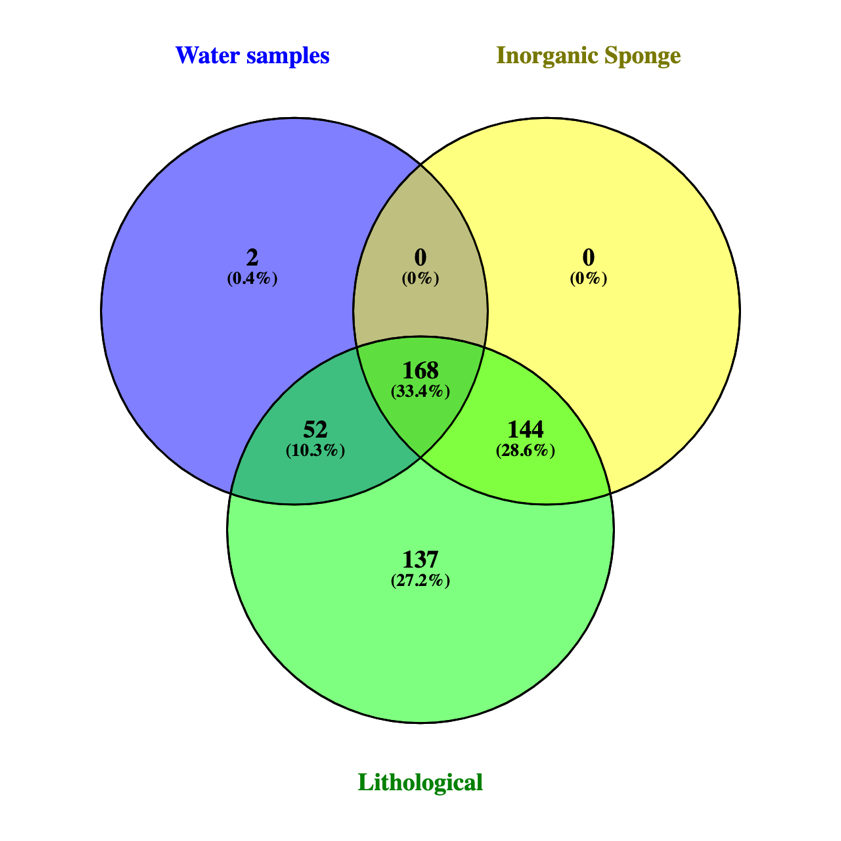
**

**Fig. S6:** Venn diagrams of OTU overlap between substrate types across all Incubations (Left) 4 categories of lithological substrates displayed. The carbonate bin contains the most unique OTUs but also has the highest number of samples. Overall, 375/520 (72%) shared across all the lithological substrates. (Right) Venn’s diagram of carbonate bin vs. water and sponge samples. Sponge samples contain no unique OTUs and water samples contain only 2, indicating that although some cells were more abundant in the planktonic fraction, most cells can be found attached to rocks. This is in agreement with evidence that rock-associated microorganisms outnumber planktonic cells by a factor of 10-10^3^.

Additional supplementary tables:

Table S5: Sequencing sample metadata

Table S6: OTU table with taxonomy and reference sequences
